# Targeting SRSF6 to Enhance Cisplatin Sensitivity by Modulating Redox Balance via NFE2L1 exon 4 Splicing in ESCC

**DOI:** 10.7150/ijbs.131590

**Published:** 2026-06-10

**Authors:** Xinyu He, Jialuo Xu, Lina Duan, Jing Ma, Dan Gao, Yifei Xie, Dengyun Zhao, Jialin Liu, Jimin Zhao, Fangfang Liu, Mee-Hyun Lee, Myoung Ok Kim, Zigang Dong, Yanan Jiang, Kangdong Liu

**Affiliations:** 1 State Key Laboratory of Metabolic Dysregulation & Prevention and Treatment of Esophageal Cancer; School of Basic Medical Sciences, Zhengzhou University, Zhengzhou 450000, Henan, China.; 2 China-US (Henan) Hormel Cancer Institute, Zhengzhou 450000, Henan, China.; 3 Tianjian Laboratory of Advanced Biomedical Sciences, Zhengzhou 450000, Henan, China.; 4 Henan International Joint Laboratory of Cancer Chemoprevention, Zhengzhou University, Zhengzhou 450000, Henan, China.; 5 Cancer Chemoprevention International Collaboration Laboratory, Zhengzhou 450000, Henan, China.; 6 Key Laboratory of Advanced Drug Preparation Technologies, Ministry of Education, School of Pharmaceutical Sciences, Zhengzhou University, Zhengzhou 450000, Henan, China.; 7 College of Korean Medicine, Dongshin University, Naju, Republic of Korea.; 8 Department of Animal Science and Biotechnology, Kyungpook National University, Sangju, Republic of Korea.

**Keywords:** SRSF6, NFE2L1, alternative splicing, cisplatin, antisense oligonucleotides

## Abstract

The mechanisms by which cancer cells survive and adapt under high levels of reactive oxygen species (ROS) remain poorly understood, especially in the context of redox homeostasis. This study reveals increased oxidative stress in esophageal squamous cell carcinoma (ESCC), with serine/arginine-rich splicing factor 6 (SRSF6) playing a crucial role in maintaining redox homeostasis. SRSF6 binds to the exonic splicing enhancer (ESE) motif in nuclear factor erythroid 2-related factor 1 (NFE2L1*)* Exon 4, preventing exon skipping and promoting the production of specific isoforms that promote ESCC cell proliferation. This interaction enhances cellular antioxidant capacity, thereby influencing redox balance. Moreover, reducing SRSF6 increases the levels of NFE2L1-S, the isoform produced by exon 4 skipping in the *NFE2L1* gene, which elevates ROS levels and induces apoptosis and ferroptosis. Notably, SRSF6 and NFE2L1 form a positive feedback loop: NFE2L1 serves as the transcription factor for SRSF6, while SRSF6 acts as the splicing factor for NFE2L1. Antisense oligonucleotides (ASOs) targeting SRSF6 significantly suppress ESCC cell growth. Importantly, inhibiting this feedback loop also enhances cisplatin (CDDP) sensitivity, increasing the therapeutic efficacy of CDDP. Our findings highlight the critical role of the SRSF6-NFE2L1 axis in redox homeostasis and tumor progression, positioning SRSF6 as a distinctive therapeutic target to improve treatment outcomes in ESCC.

## Introduction

Elevated oxidative stress is a hallmark of tumorigenesis in numerous cancers 1. In tumor cells, oxidative stress results from rapid growth and high metabolic activity, which leads to excessive production of reactive oxygen species (ROS). The accumulation of ROS can cause damage to cellular components, including DNA, proteins, and lipids, thereby threatening cell survival 2. To reduce oxidative stress, cells utilize antioxidant defense mechanisms to restore redox homeostasis. These include antioxidant molecules such as glutathione (GSH), catalase (CAT), superoxide dismutase (SOD), and peroxiredoxins (PRX), as well as transcription factors like nuclear factor erythroid 2-related factor 1 (NFE2L1) and nuclear factor erythroid 2-related factor 2 (NFE2L2), which regulate the expression of antioxidant genes by binding to antioxidant response elements (ARE) in the promoters of these genes 3,4. Studies have shown that esophageal squamous cell carcinoma (ESCC) is characterized by oncogenic activation of the NFE2L2 family, accompanied by elevated ROS levels 5. Our previous research suggested that targeting NFE2L2 to induce ROS accumulation presents a promising therapeutic strategy for ESCC 6. Understanding the molecular mechanisms that control redox homeostasis in ESCC is essential for identifying potential therapeutic strategies to disrupt this balance and inhibit cancer progression.

Abnormal regulation of alternative splicing (AS) plays a vital role in the occurrence and development of tumors, involving various tumor processes such as angiogenesis, tumor invasion and metastasis, and immune dysfunction 7. The serine/arginine-rich (SR) protein family plays a crucial role in splicing regulation 8. Abnormal expression of SR proteins has been observed in various cancers, where they modulate splicing events that facilitate proliferation and survival 9. Accumulated evidence indicates alterations in SR protein expression contribute to tumor progression by disrupting splicing events 10-13. Phosphoproteomic data indicate that SR proteins are usually highly phosphorylated in ESCC 14-16. Serine/arginine-rich splicing factor 6 (SRSF6), also known as SRp55, is a member of the SR protein family and is considered a key regulator of. Recently, compelling evidence has demonstrated the overexpression of SRSF6 in human skin cancer. Moreover, transgenic mice with elevated levels of this gene exhibit pronounced skin hyperplasia and aberrant splicing 17. In colorectal cancer cells, downregulation of SRSF6 can significantly inhibit the migration of cancer cells *in vitro* and *in vivo* 12. These findings suggest that SRSF6 is an oncogene and potentially contributes to aberrant splicing in various cancer types. However, the expression pattern and functional role of SRSF6 in ESCC remain unknown.

This dynamic regulation of ROS and antioxidant responses is critical for tumor cell survival and growth. Despite the significant advances in understanding redox regulation, several key questions remain unresolved in the context of cancer biology. One major question is how specific splicing factors, like SRSF6, influence redox homeostasis and contribute to tumor progression. While the role of NFE2L1 in maintaining redox balance is well established, the mechanisms by which alternative splicing of NFE2L1 and other genes related to oxidative stress are regulated in cancer cells are not fully understood. Furthermore, how dysregulation of this process in cancer cells affects drug sensitivity and impacts therapeutic outcomes has not been adequately explored. Cisplatin (CDDP) is one of the most effective chemotherapy agents for ESCC, but its clinical success is hindered by the development of resistance, which significantly reduces its effectiveness in treating patients 18. Enhancing CDDP sensitivity is a major challenge in ESCC therapy, making it critical to explore strategies that could sensitize tumors to CDDP and improve patient outcomes.

Our research reveals a novel mechanism through which SRSF6 promotes cell proliferation in ESCC by regulating AS to enhance antioxidant defense and restore redox homeostasis. Specifically, SRSF6 prevents the skipping of exon 4 in the *NFE2L1* gene, resulting in the production of the NFE2L1-L isoform, which strengthens the binding of NFE2L1 to the ARE. This binding upregulates genes involved in the cellular response to ROS, thereby counteracting ROS-induced damage and preventing apoptosis, ultimately promoting cell survival and proliferation. Furthermore, our findings highlight the clinical significance of targeting SRSF6 to enhance CDDP sensitivity. By disrupting the SRSF6-mediated regulation of NFE2L1 splicing, we observed increased ROS levels, enhanced apoptosis, and signs of ferroptosis. These findings suggest that inhibiting SRSF6 sensitizes ESCC cells to CDDP by promoting both apoptosis and ferroptosis. This study uncovers the crucial role of SRSF6 in maintaining redox homeostasis and promoting tumor progression, while also providing a potential therapeutic strategy to enhance CDDP sensitivity and improve treatment outcomes in ESCC patients.

## Materials and Methods

### Cell lines

The immortalized human esophageal epithelial cell line SHEE was kindly provided by Professor Enmin Li. Human ESCC cell lines KYSE30, KYSE70, KYSE140, KYSE150, KYSE410, KYSE450, and KYSE510 were purchased from the Cell Bank of the Chinese Academy of Sciences (Shanghai, China). The cell lines were also verified by STR analysis and were mycoplasma-free. At 37 °C and 5% CO_2_, ESCC cells were grown in RPMI-1640 medium with 10% fetal bovine serum (FBS).

### Immunohistochemistry (IHC)

IHC staining was conducted as previously reported 6. Tumors and adjacent tissues from 60 ESCC cases in the tissue microarray were provided by the Pathology Department of Henan Cancer Hospital, Henan, China. SRSF6 protein expression was detected using conventional immunohistochemistry techniques. Whole-slide images were analyzed using StrataQuest Analysis software (TissueGnostics, Austria), an automated digital pathology platform for quantitative image analysis 19. The software automatically performed cell segmentation and identification of SRSF6-positive cells based on DAB staining signals. All tissue samples were analyzed using the same analysis parameters and threshold settings. The percentage of SRSF6-positive cells was calculated for each tissue sample and used as the quantitative index of SRSF6 expression. Cases were subsequently classified into high- and low-expression groups according to the median percentage of positive cells.

### Western blot

RIPA buffer was used to lyse the tissues or cells (Solarbio, China; Catalog No. R0010). For the Western blot, the same quantities of total protein were loaded. Resolved proteins were subsequently transferred to PVDF membranes (Millipore, USA) following SDS-PAGE. The membranes were blocked in 5% skim milk for 1 h at room temperature (RT), followed by 1 h at RT or an overnight incubation with primary antibodies (Table S1), including SRSF6 (sc-57954), NFE2L1 (sc-515360), G6PD (sc-373886), GCLC (sc-166356), GCLM (sc-55586), and GSR (sc-133245) (all from Santa Cruz Biotechnology, Dallas, TX, USA); GPX4 (ab125066) and Ki67 (ab15580) (Abcam, UK); GAPDH (60004-1-Ig, Proteintech, USA). After 2 h of secondary antibody incubation, we recovered the secondary antibody by washing the membrane with Tris-buffered saline (TBS) solution 3 times for 5 min each time and then utilized a chemiluminescence device to expose it using a Tanon 5200 Multi imaging system (Tanon, China) 20.

### Knockdown and knockout of SRSF6 in ESCC cells

Lentiviral vectors were used to achieve both knockdown and knockout of SRSF6 in ESCC cells. For knockdown, short hairpin RNA (shRNA) sequences targeting human SRSF6 (Table S2) were inserted into the pLKO.1 vector. For knockout, two SRSF6-specific sgRNAs (Table S3) were designed using the CHOPCHOP online tool. The corresponding pLKO.1-shSRSF6 or CRISPR/Cas9 sgRNA constructs, together with the packaging plasmids pMD2.G and psPAX2, were transfected into HEK293T cells using jetPRIME according to standard transfection protocols. Viral supernatants were collected at 24, 48, and 72 h post-transfection through a 0.22 μm filter, mixed with 8 μg/mL polybrene, and used to infect KYSE70 and KYSE450 ESCC cells. After infection, KYSE70 cells were selected with 2 μg/mL puromycin for 72 h, and KYSE450 cells with 1 μg/mL puromycin for 72 h. The efficiency of SRSF6 knockdown or knockout was evaluated by Western blot analysis.

### MTT assays

Trypsinized cells were counted and then plated into 96-well plates containing 200 µL of culture medium at a density of 1.5 × 10^3^ cells per well. Cell viability experiments were performed using the 3-(4,5-dimethylthiazol-2-yl)-2,5-diphenyltetrazolium bromide (MTT) reagent (Sigma-Aldrich, USA; Cat. No. 475989) following incubation for 24, 48, 72, and 96 h. The MTT reagent was directly added to each well, followed by a 2-hour incubation period. Subsequently, the supernatant was carefully aspirated, and the reaction was stopped by adding DMSO. The absorbance at 490 nm was then measured using a reader system. The ferroptosis inhibitor Ferrostatin-1 and pan-caspase inhibitor Z-VAD were purchased from MedChemExpress (MCE, USA) and used in the MTT assay. Each experiment was performed with three replicate wells per condition to ensure data reliability.

### Clone formation assay

The cells were dissociated into single-cell suspensions using 0.25% trypsin. Six-well plates were utilized, each containing an average of 200 cells per well. The cells were incubated at 37°C with 5% CO_2_ for two weeks prior to being rinsed thrice with PBS and subsequently fixed in methanol for 20 min at room temperature (RT). Following fixation, the cells were stained with 0.1% crystal violet dye for a period of 30 min, and photographs of colonies were captured. Colony-forming efficiency was calculated using the following formula: colony-forming efficiency = number of colonies counted / number of cells plated × 100%. Each experiment was performed with three replicate wells per condition to ensure data reliability.

### Anchorage-independent cell growth assay

The 0.3% agar was then supplemented with ESCC cells (8 × 10^3^ cells per well) suspended in a complete RPMI-1640 medium containing L-glutamine (VivaCell, China; Catalog No. C3010-0500) with 10% Fetal bovine serum (VivaCell, China; Catalog No. C04001-500) and 1% Penicillin-Streptomycin solution (Meilunbio, China; Catalog No. MA0110). Then, 0.6% agar was put into each of the six wells in the plate. The cultures were kept at 37°C in an incubator with 5% CO_2_ for 2-3 weeks, and colonies were counted using a high-content imaging system (In Cell Analyzer 6000, GE Healthcare) under a microscope 21. Each experiment was performed with three replicate wells to ensure data reliability.

### Cell-derived xenograft mouse model (CDX)

The experimental mice used were female BALB/c nude mice, aged 6-8 weeks, purchased from Charles River Laboratories (Beijing, China). The mice were raised in the animal laboratory of the Henan Collaborative Innovation Center for Cancer Chemical Prevention in a SPF environment, with a temperature maintained at 21-25°C and humidity held at 45%-50%. Mice were randomly allocated to three groups (n = 8) and subcutaneously injected into the right flank with 1 × 10⁶ KYSE450 or KYSE70 cells that were either control, sgSRSF6#3, or sgSRSF6#5 cells. After 2 weeks, the volume of the tumors was measured every 3 days using the formula:(short diameter)^2^ × (long diameter)/2. At the experimental endpoint, mice were euthanized, and tumors were excised and weighed.

### Next-generation RNA-sequencing analyses

Sequencing data were filtered using SOAPnuke by applying the following criteria: (1) reads containing sequencing adapters were removed; (2) reads in which the proportion of low-quality bases (quality ≤ 15) exceeded 20% were removed; (3) reads in which the proportion of unknown ('N') bases exceeded 5% were removed. Afterwards, clean reads were obtained and stored in FASTQ format. Subsequent analyses and data mining were performed using the Dr. Tom Multi-omics Data Mining System (https://biosys.bgi.com). Gene expression levels were estimated using RSEM (v1.3.1). Heatmaps were generated using the pheatmap package (v1.0.8) based on gene expression variations among samples.

### Bioinformatics analysis

To explore the molecular mechanisms underlying the observed phenotypes, we performed comprehensive bioinformatics analyses. Gene Set Enrichment Analysis (GSEA) was conducted to identify enriched pathways and biological processes associated with differential gene expression, using the GSEA software (v4.3.2, Broad Institute) with hallmark and KEGG gene sets from the Molecular Signatures Database (MSigDB). The ranking metric used was the signal-to-noise ratio, and the analysis applied the phenotype-based permutation approach with 1000 permutations. Gene Ontology (GO) and Kyoto Encyclopedia of Genes and Genomes (KEGG) enrichment analyses were performed using the SangerBox platform (http://www.sangerbox.com/home.html) with default parameters. Differentially expressed genes (DEGs) were input to identify significantly enriched biological functions and signaling pathways, based on hypergeometric testing and multiple testing correction (FDR < 0.05) 22.

### Analysis of alternative splicing events

To assess alternative splicing events, we utilized rMATS to analyze RNA-seq data. The software quantified and compared five types of splicing events: exon skipping (SE), alternative 3' splice site (A3SS), alternative 5' splice site (A5SS), mutually exclusive exons (MXE), and retained intron (RI). The rMATS analysis incorporated biological replicates and applied a minimum read count threshold of 10 reads per event. A delta-PSI cutoff of |ΔPSI| ≥ 0.1 was used to define significant splicing changes, with an FDR < 0.05 for multiple testing correction.

### Reverse transcription and PCR analysis

Total RNA was isolated from cultured cells using TRIzol reagent (Ambion, USA). Reverse transcription was carried out using the HiScript III RT SuperMix for qPCR Kit (Vazyme, Nanjing, China; Cat. No. R323). For quantitative real-time PCR (qPCR), 2 μg of total RNA was reverse transcribed, and amplification was performed on a StepOnePlus™ real-time PCR system (Thermo Scientific, USA) with SYBR® Premix Ex Taq™ II (Takara, Japan). *ACTB* served as the internal control, and all reactions were run in triplicate. The primer sequences used for qPCR are provided in Table S4. For reverse transcription PCR (RT-PCR), 1 μg of total RNA was reverse transcribed. PCR amplification was performed in a 25 μL reaction volume containing 0.5 μg of the reverse transcription product, using 2 × Phanta Flash Master Mix (Dye Plus) (Vazyme, Nanjing, China), with 30 amplification cycles. The primers used for RT-PCR are listed in Table S5.

### Chromatin immunoprecipitation assay (ChIP)

ChIP assays were performed to investigate the binding of NFE2L1 to the promoter regions of SRSF6, glutathione peroxidase 4 (GPX4), glutamate-cysteine ligase catalytic subunit (GCLC), and glucose-6-phosphate dehydrogenase (G6PD) in KYSE70 and KYSE450 cell lines. Cells were cultured to 75% confluence in 15 cm plates and crosslinked with 1% formaldehyde at 37°C for 10 min to preserve protein-DNA interactions, followed by quenching with 125 mM glycine in PBS for 2 min at RT. After washing with PBS, the cells were pelleted and lysed to isolate the nuclei. The chromatin was then sheared into 200-500 bp fragments using an Ultrasonic Cell Disruption System. Immunoprecipitation was performed using an NFE2L1 antibody, with IgG as a control. DNA-protein crosslinks were then reversed, and the DNA was purified for analysis. ChIP-qPCR was conducted using specific primers for GPX4, GCLC, G6PD, and SRSF6 promoter regions to assess NFE2L1 binding. Primer sequences used for ChIP-qPCR are listed in Table S6. Enrichment of the promoter regions in the immunoprecipitated chromatin was quantified relative to input DNA.

### Minigene assay

The *NFE2L1* gene's genomic DNA fragment, which spans exons 3 through 5, was cloned into the pcDNA3.1 (+) plasmid. The NFE2L1 splicing minigene assay was carried out in 293T cells. The constructed pcDNA3.1-NFE2L1-minigene plasmids and overexpressing SRSF6-pLVX-IRES-Puro-3×Flag plasmids were co-transfected into 293T cells. Cells were collected 48 h after transfection for RNA extraction and reverse transcription, followed by RT-PCR to examine spliced isoforms. They were then resolved by agarose gel electrophoresis.

### RNA-binding protein immunoprecipitation (RIP) assay

The cells were collected and resuspended in an equal volume of IP lysis buffer, and then were lysed on ice for 40 min. The cells were harvested, and their concentration was determined. The magnetic beads were thoroughly washed and resuspended, then collected using a magnetic separator. Each EP tube was incubated with 3.5 μg of FLAG tag antibody and 400 μL of PBST at 4 °C for 2 h. Lysis buffer containing RNase inhibitors was added to a final volume of 0.8 mL, and the samples were incubated at 4 °C for 3 h. Following incubation, the cells were washed with 0.5 mL of PBST. Total RNA was extracted using TRIzol reagent. Briefly, 1 mL TRIzol was added to each sample, followed by vortexing and incubation on ice for 5 min. After chloroform addition and phase separation, the aqueous phase was collected. RNA was precipitated with isopropanol, centrifuged (15,000 rpm, 30 min, 4°C), and the pellet was washed with 75% ethanol, air-dried, and dissolved in 15 μL RNase-free water. The resulting RNA was then analyzed by reverse transcription and RT-PCR.

### Dual-luciferase reporter assay

Dual-luciferase reporter assays were conducted to evaluate the binding interaction of NFE2L1 with both the SRSF6 promoter region and a general ARE sequence. The SRSF6 promoter sequence, containing either the wild-type or mutant version of the predicted NFE2L1 binding site, was cloned into a luciferase reporter plasmid and co-transfected with an NFE2L1 expression vector. Additionally, six copies of the ARE sequence were inserted into the pGL4.19 [lucCP/Neo] plasmid (Promega, E674A) to generate the ARE construct, which was co-transfected with either NFE2L1-S or NFE2L1-L isoforms. The sequences of all cloned fragments are provided in Table S7. For all transfections, the reaction system per well included 0.5 μg of the target plasmid, 25 ng of Renilla luciferase plasmid for normalization, 50 μL of jetPRIME buffer, and 2 μL of jetPRIME reagent. After 48 h, cells were harvested, and luciferase activity was measured using a dual-luciferase reporter assay system, with Renilla luciferase activity used to normalize transfection efficiency. Sample preparation and fluorescence detection followed previously described protocols 6. Each experiment was performed with three replicate wells per condition to ensure data reliability.

### Electrophoretic mobility shift assay (EMSA)

The direct transcript activation of *NFE2L1* was examined by EMSA. The ARE probe used for EMSA was generated from complementary oligonucleotides, as detailed in Table S8. This oligomer was biotin-labeled at the end (Sangon Biotech, Shanghai, China). It was then annealed with its antisense oligomer to make a double-stranded probe. Binding reactions were carried out according to the manufacturer's instructions using a Lightshift EMSA Optimization and Control kit (Thermo Scientific, 20148X). On a 6% non-denaturing polyacrylamide gel, the DNA-protein complex was dissociated at 100 V in Tris-Borate-EDTA buffer (45 mM Tris-borate, 1 mM EDTA, pH 8.3). A nylon membrane (Beyotime, FFN10) was electro-transferred with DNA-protein complexes, and the membrane's DNA was crosslinked for 10 min using a UV-light crosslinking device with 254 nm bulbs. Blots were detected using the Chemiluminescent Nucleic Acid Detection Module (Thermo Scientific, 89880) and visualized with a Tanon 5200 Multi Imaging System (Tanon, China).

### Intracellular ROS production assays

The fluorescent molecule 2′,7′-dichlorofluorescein (DCF), generated upon peroxide-dependent intracellular oxidation of 2′,7′-dichlorodihydrofluorescein diacetate (DCFH-DA), was used to monitor ROS levels. After receiving the treatments, ESCC cells were incubated at 37°C for 30 min with added fresh media containing 10 μM DCFH-DA (Sigma-Aldrich). PBS was used to wash the cells twice, and trypsin was used to digest them. Additionally, NovoCyte flow cytometry (ACEA Biosciences, USA) and IN Cell Analyzer 6000 photography (GE Healthcare, USA) were used to measure ROS levels.

### Detection of malondialdehyde (MDA) level

The MDA level was measured using the lipid peroxidation MDA assay kit (#S0131, Beyotime) according to the manufacturer's instructions. In brief, KYSE70 and KYSE450 cells were washed 4 times with PBS before being lysed. The lysates were centrifuged for 10 min at 12000 rpm. The supernatant was then gathered for MDA detection. Finally, a microplate reader (Thermo Fisher Scientific, USA) recorded the absorbance at 532 nm, and the MDA level was standardized by the protein content in each sample. Each experiment was performed with three replicate wells per condition to ensure data reliability.

### GSH and GSSG assays

The contents of GSH and GSSG were determined according to the manufacturer's procedure using the GSH and GSSG assay kit (Beyotime Biotechnology, China). A protein removal agent was applied after the cells were rinsed with PBS. Liquid nitrogen and a 37 °C water bath were used to freeze and thaw the samples twice. The cells were centrifuged at 12,000 ×g for 15 min at 4°C. GSH and GSSG were determined in the supernatant. The absorbance at 412 nm was measured using a microplate reader (Thermo Fisher Scientific, USA). Each experiment was performed with three replicate wells per condition to ensure data reliability.

### Flow cytometric analysis of cell apoptosis

According to the manufacturer's instructions, KYSE70 and KYSE450 cells were stained using an Annexin V-FITC/PI apoptosis kit (MultiSciences, China). KYSE70 and KYSE450 cells were resuspended in PBS and stained with FITC-Annexin V and propidium iodide (PI). Flow cytometry analysis was performed using a NovoCyte flow cytometer (ACEA Biosciences, USA) to evaluate the results. The gating strategy involved using forward scatter (FSC) vs. side scatter (SSC) to exclude debris and identify single cells. Early-stage apoptotic cells were identified as Annexin V-positive and PI-negative, while late-stage apoptotic or necrotic cells were both Annexin V-positive and PI-positive. Viable cells were defined as negative for both Annexin V and PI. Each condition was performed with three technical replicates to ensure data reliability.

### Drug synergy analysis

Drug combination synergy was evaluated using the SynergyFinder 2.0 web tool (https://synergyfinder.fimm.fi), which integrates multiple synergy scoring models, including Bliss, Loewe, HSA, and ZIP. Dose-response matrix data were uploaded, and the ZIP score was used to assess synergistic interactions between ASO-1154 and CDDP.

### ASO treatment in xenograft mouse models

To investigate the effects of ASO treatment alone and in combination with CDDP on tumor growth, two independent *in vivo* experiments were conducted using a KYSE450 xenograft mouse model. In the ASO monotherapy experiment, 6-week-old BALB/c nude mice were subcutaneously injected with 5 × 10⁶ KYSE450 cells. Once tumors reached approximately 50 mm³, mice were randomly assigned into three groups (n = 8 per group): ASO-NC (negative control), ASO-926, and ASO-1154. ASO-926 (5'-TACTCATCCTTAGATCTGC-3') and ASO-1154 (5'-TAATCTCTGGAACTCGACC-3') were synthesized with 2'-O-methyl (2-OMe) and phosphorothioate (PS) modifications to enhance stability and binding affinity. These ASOs were administered intratumorally at a dose of 1 OD per mouse, dissolved in sterile PBS, and delivered every five days. In the ASO-CDDP combination experiment, another cohort of mice with tumors of similar size was randomly divided into three groups (n = 8): ASO-NC + CDDP, ASO-1154 + CDDP, and CDDP alone. CDDP was administered intraperitoneally at a dose of 3 mg/kg every three days, while ASO-1154 was delivered intratumorally at the same dosing schedule as in the monotherapy experiment. Tumor growth was monitored throughout the study, and at the experimental endpoint, all mice were euthanized for tumor excision, imaging, and weighing. Tumor weights were compared among groups to assess the effects of ASO-1154 alone and in combination with CDDP on tumor suppression. The tumor volume was calculated by (short diameter)^2^ × (long diameter)/2. The mice were euthanized at the end of the experiments, and the subcutaneous tumor tissues were excised for weighing and photography.

### Statistical analysis

SPSS 21.0 was used to conduct all statistical analyses, and quantitative results are shown as the mean ± SD. One-way analysis of variance (ANOVA) or Student's *t*-test was used to compare significant differences. *P < 0.05, **P < 0.01, and ***P < 0.001 were used to show significance.

## Results

### SRSF6 is a key regulator of redox homeostasis and is associated with prognosis in ESCC

**Figure 1:**
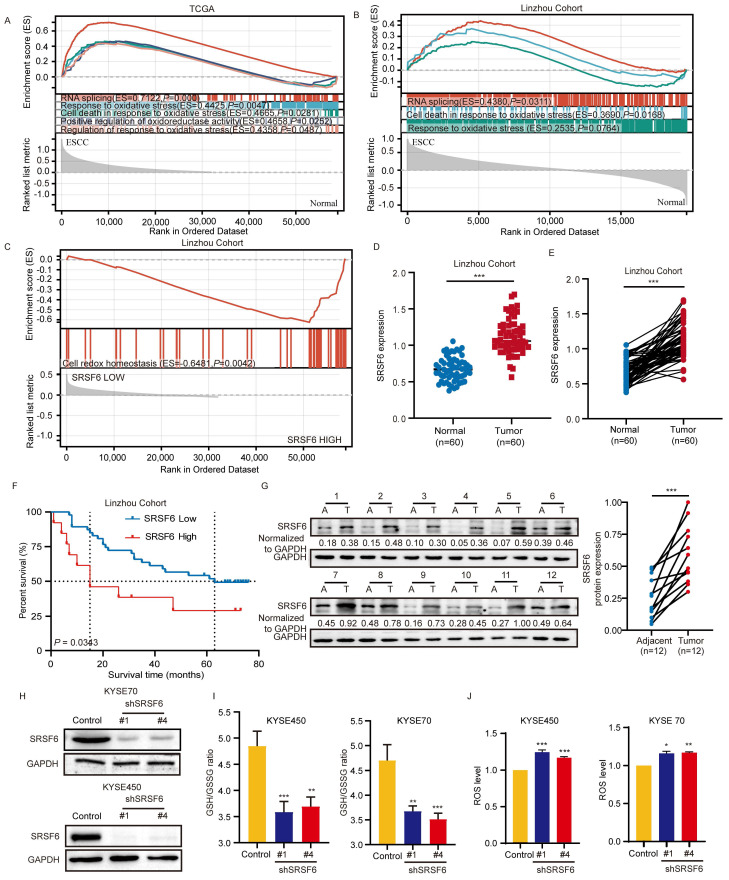
**SRSF6 is a key regulator of redox homeostasis and is associated with prognosis in ESCC. A, B,** GSEA was performed on both the TCGA ESCC dataset (**A**) and the Linzhou cohort (**B**) to compare gene expression profiles between ESCC and normal tissue groups. In both datasets, pathways related to RNA splicing, response to oxidative stress, and cell death in response to oxidative stress were identified as enriched in the ESCC group. **C,** GSEA was performed in the Linzhou cohort comparing the high and low SRSF6 expression groups; the gene set 'cell redox homeostasis' is shown. **D, E,** Expression levels of the SRSF6 protein were analyzed via proteomics approaches in unmatched (**D**) and matched (**E**) tumors, and adjacent normal tissues were obtained from a cohort of 60 patients with ESCC.** F,** Kaplan-Meier analysis of SRSF6 of ESCC patients from proteomics analysis. **G**, Clinical esophageal samples, including adjacent normal (A) and tumor (T) tissues, were collected to assess SRSF6 expression levels. **H,** The protein levels of SRSF6 knockdown in KYSE70 and KYSE450 cells of ESCC were detected by Western blot assay.** I,** GSH/GSSG ratios of KYSE70 and KYSE450 cells were measured after SRSF6 knockdown (n = 3). **J,** ROS levels were measured by flow cytometry in KYSE70 and KYSE450 cells after SRSF6 knockdown, and quantified as relative ROS levels (n = 3). All the data are presented as the mean ± SD. Data in panel D were analyzed by independent two-sample t-test; panels E and G by paired t-test; panels I and J by one-way ANOVA; panel F by Kaplan-Meier analysis with log-rank test. Asterisks indicate statistical significance (*P < 0.05, **P < 0.01, ***P < 0.001).

Through the analysis of TCGA data on ESCC, it was observed that pathways related to RNA splicing, response to oxidative stress, cell death in response to oxidative stress, positive regulation of oxidoreductase activity, and regulation of response to oxidative stress are significantly upregulated in ESCC (Fig. 1A). These findings indicate that splicing processes and oxidative stress responses are aberrantly activated in ESCC, potentially contributing to tumor progression. Through the analysis of our own sequencing data generated from the Linzhou ESCC cohort (ProteomeXchange ID: PXD035562), similar upregulation of pathways related to RNA splicing, cell death in response to oxidative stress, and response to oxidative stress was observed (Fig. 1B). These findings further support the observation that these pathways are aberrantly activated in ESCC, consistent with the results from the Linzhou cohort. Through the analysis of alternative splicing regulators in the Linzhou cohort, we identified several splicing factors that exhibit significant correlations with the redox homeostasis pathway (Fig. S1A). Among them, SRSF6 showed a notably strong positive correlation, suggesting its potential role in regulating redox balance in tumor cells (Fig. 1C). Our comprehensive proteomic profiling of 60 ESCC patients revealed significant overexpression of the splicing regulator SRSF6 14, specifically in tumor tissues compared to adjacent tissues (Fig. 1D) and paired samples (Fig. 1E). Importantly, high expression of SRSF6 correlated with poor prognosis (Fig. 1F). Furthermore, we assessed protein levels of SRSF6 in paired ESCC tissues and found its levels were markedly higher in tumor tissue (T) than in adjacent (A) tissues (Fig. 1G).

To assess the impact of SRSF6 in ESCC, we analyzed the protein levels of SRSF6 in various ESCC cell lines (KYSE30, KYSE70, KYSE140, KYSE150, KYSE410, KYSE450, and KYSE510) as well as immortalized esophagus epithelial cell (SHEE) using Western blot. Compared to SHEE, the protein levels of SRSF6 were significantly higher in ESCC cells, especially in KYSE450 and KYSE70 cells, while comparatively lower in KYSE150 cells (Fig. S1B). To further validate the role of SRSF6 in redox metabolism, SRSF6 knockdown cell lines were established in KYSE70 and KYSE450 cells using the shRNA system (Fig. 1H). Subsequently, the GSH/GSSG ratio was measured in these SRSF6 knockdown and control cells, revealing a significant reduction in the ratio following SRSF6 knockdown (Fig. 1I). This indicates a decrease in the cellular reduction potential and an increase in oxidative stress, further supporting the role of SRSF6 in maintaining redox balance in ESCC. In addition, the ROS levels in SRSF6 knockdown and control cells were measured using flow cytometry. The results showed a significant increase in ROS levels in the SRSF6 knockdown cells, further confirming that the loss of SRSF6 leads to elevated oxidative stress in ESCC cells (Fig. 1J and Fig. S1C). Moreover, overexpression of SRSF6 in both KYSE70 and KYSE450 cells significantly reduced MDA levels, a marker of lipid peroxidation, compared to the control group (Fig. S1D), reinforcing the involvement of SRSF6 in regulating oxidative stress and lipid peroxidation.

### SRSF6 regulates alternative splicing and exon 4 skipping of transcription factor NFE2L1 in ESCC

**Figure 2:**
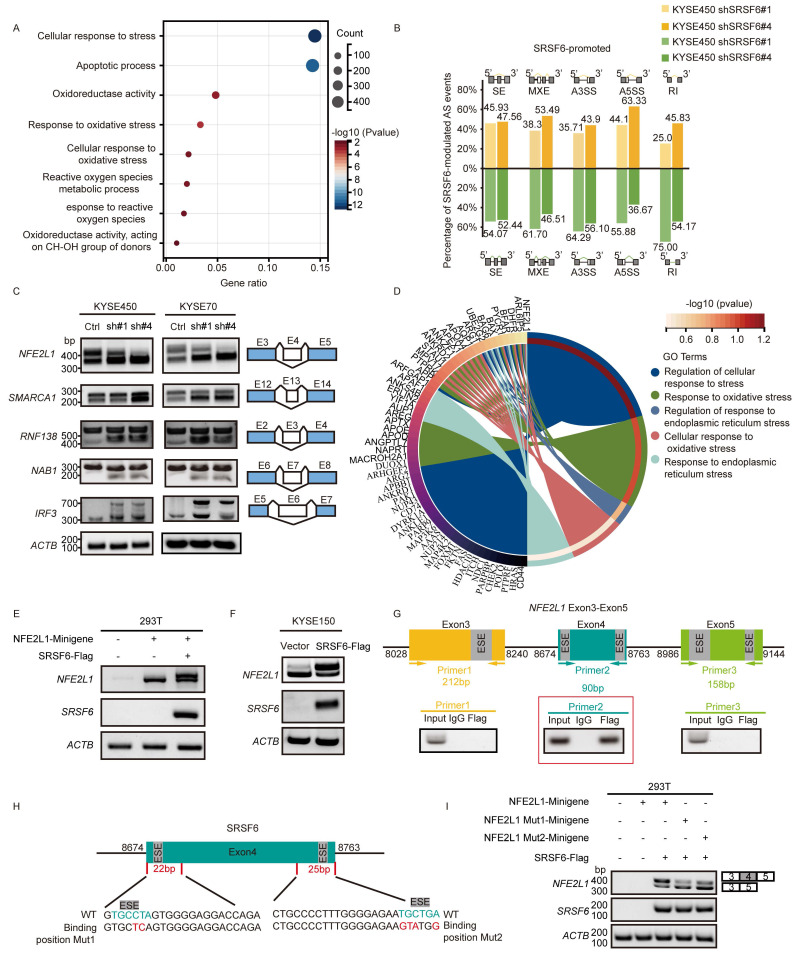
**SRSF6 regulates alternative splicing and exon 4 skipping of transcription factor NFE2L1 in ESCC. A,** Differential gene expression analysis was performed by comparing KYSE450 cells transfected with shSRSF6 to those transfected with control. Gene set functional enrichment analysis was conducted using the GO annotations of genes. **B,** The proportion of SRSF6 down-regulated genes in five types of splicing events in ESCC was detected by transcriptional sequencing. **C,** RT-PCR analysis was performed on randomly selected SRSF6-regulated splicing events. **D,** The enrichment results of differentially alternatively spliced genes in GO biological processes, visualized in a circular plot. **E,** Minigene report analysis design for detection of NFE2L1 exon 4 splicing. The splicing changes of the NFE2L1 transcript after co-transfection of SRSF6-Flag and NFE2L1-minigene into 293T cells were analyzed by RT-PCR. **F,** RT-PCR analysis of splicing changes of NFE2L1 transcripts in control and SRSF6-overexpressed cells. **G,** NFE2L1 Exon 3-5 Primers were designed at different locations, and SRSF6 binding to exon 4 of NFE2L1 was determined using RIP-PCR. **H,** Design of mutations (Mut1/Mut2) targeting the predicted SRSF6-binding motifs within NFE2L1 exon 4. **I,** Splicing changes of NFE2L1 transcripts after mutation of the exon 4 binding site of NFE2L1-minigene were detected by RT-PCR.

Splicing factors are essential in the pre-mRNA splicing process 23. After identifying SRSF6 as a critical splicing factor involved in both ESCC proliferation and redox regulation, we conducted RNA sequencing to explore its role in both mRNA splicing and alternative splicing events, as well as to examine differential gene expression. This dual approach allows us to investigate how SRSF6 influences splicing dynamics while also identify which pathways are affected by gene changes, providing a comprehensive understanding of its regulatory functions. Initially, we analyzed the differentially expressed genes following SRSF6 knockdown. In the shSRSF6#1 and shSRSF6#4 groups, compared to the control group, 2342 genes were upregulated, and 2132 genes were downregulated in the shSRSF6#1 group, while 2606 genes were upregulated and 2323 genes were downregulated in the shSRSF6#4 group (Fig. S2A). Additionally, a Venn diagram analysis was performed to identify the intersection of differentially expressed genes between the shSRSF6#1 and shSRSF6#4 groups. This analysis revealed 2026 commonly upregulated and 1822 commonly downregulated transcripts following SRSF6 depletion (Fig. S2B). GO enrichment analysis was performed on the commonly differentially expressed genes to both the shSRSF6#1 and shSRSF6#4 groups, revealing significant enrichment in pathways related to cellular response to stress, apoptosis, oxidoreductase activity, response to oxidative stress, and ROS metabolic processes (Fig. 2A). These findings highlighted the pivotal role of SRSF6 in modulating key cellular functions, particularly those involved in oxidative stress responses and the regulation of ROS, suggesting that SRSF6 is crucial for maintaining redox balance within the cell.

Furthermore, the analysis of alternative splicing events in KYSE450 cells subjected to knockdown of SRSF6 using shSRSF6#1 and shSRSF6#4, along with a control, revealed five distinct categories of AS (Fig. S2C) 10. Utilizing the differential splicing analysis tool rMATS, a significant number of splicing events were identified, with a FDR ≤ 0.05. Among these splicing events, exon skipping was identified as the predominant category (51%), followed by A3SS (18%), A5SS (14%), RI (7%), and MXE (10%) in the control group. In contrast, SE accounted for 58% and 51%, A3SS was 19% and 17%, A5SS was 15% and 13%, MXE was 1% and 11%, and RI was 7% and 8% in shSRSF6 #1 and shSRSF6 #4, respectively (Fig. S2D). After the knockdown of SRSF6, the shSRSF6 #1 and #4 variants exhibited increases in various splicing events: 45.93% and 47.56% of SE, 38.30% and 53.49% of MXE, 35.71% and 43.90% of A3SS, 44.12% and 63.33% of A5SS, and 25% and 45.83% of RI, respectively. In comparison to the control group, they also demonstrated a suppressive effect on splicing events: 54.07% and 52.44% of SE, 61.70% and 46.51% of MXE, 64.29% and 56.10% of A3SS, 55.88% and 36.67% of A5SS, and 75% and 54.17% of RI (Fig. 2B), respectively. These findings strongly support the role of SRSF6 in regulating the splicing of target genes and causing exon skipping.

Subsequently, alternative splicing events were validated by selecting five genes, with NFE2L1 chosen due to its significant role, and the remaining genes selected randomly. Our results demonstrated that silencing SRSF6 led to the skipping of exon 4 of *NFE2L1*, exon 13 of SWI/SNF-related, matrix-associated, actin-dependent regulator of chromatin, subfamily A, member 1 (SMARCA1), exon 3 of RING finger protein 138 (RNF138), exon 7 of NGFI-A-binding protein 1 (NAB1), and exon 6 of interferon regulatory factor 3 (IRF3) (Fig. 2C). Additionally, GO enrichment analysis was conducted on the genes corresponding to differential alternative splicing events, resulting in significant enrichment in pathways such as regulation of cellular response to stress, response to oxidative stress, and others. In the circular plot, individual genes and pathways are represented by their respective *P*-values. Among these, NFE2L1 stands out with the lowest *P*-value, indicating its potential importance within these enriched pathways (Fig. 2D). These findings underscore the crucial role of alternative splicing in stress response pathways in ESCC, with NFE2L1 emerging as a key regulatory factor influenced by the silencing of SRSF6, highlighting the potential of targeting splicing regulators like SRSF6 for therapeutic interventions in ESCC.

Next, we searched the National Center for Biotechnology Information (NCBI) database and discovered two distinct transcript variants of *NFE2L1*. The first variant, NM_003204.3, denoted as *NFE2L1-L* and encompassing exon 4, represents the full-length transcript variant. Conversely, the second variant, identified as NM_001330262.2, is referred to as *NFE2L1-S* and constitutes a shorter transcript that excludes exon 4. Furthermore, the RNA-sequencing results showed that SRSF6 inhibited the exon 4 skipping of *NFE2L1*, thereby establishing it as a promising candidate gene. Notably, NFE2L1 plays a vital role in regulating the balance of intracellular redox levels by controlling the transcription of different groups of target genes. To gain further insights into the modulation of exon 4 skipping in NFE2L1 transcripts by SRSF6, we transfected the NFE2L1 minigene plasmid into 293T cells. NFE2L1-L isoform expression was upregulated by co-transfecting the NFE2L1 minigene with SRSF6-Flag plasmids (Fig. 2E). Additionally, it was observed that the overexpression of SRSF6 enhanced the expression of NFE2L1-L isoform by inhibiting the skipping of *NFE2L1* exon 4 (Fig. 2F). These findings suggest that SRSF6 inhibits the skipping of *NFE2L1* exon 4, boosting the expression of full-length *NFE2L1* in ESCC cells.

SRSF6 has the characteristic of binding with the ESE motif 24. Subsequently, we further investigated whether SRSF6 directly interacts with *NFE2L1* mRNA through specific binding sites and predicted potential SRSF6 binding sites in exon 3, exon 4, and exon 5 of NFE2L1 using the ESE finder (http://exon.cshl.edu/ESE/). Based on the prediction sites of ESE, three pairs of different primers targeting exons 3-5 of *NFE2L1* were designed to check the binding position of NFE2L1 by SRSF6. The results of the RIP assay demonstrated that primer 2, but not primer 1 or primer 3, had a positive product (Fig. 2G), suggesting that SRSF6 could bind to exon 4 of *NFE2L1*. Therefore, we speculated that SRSF6 directly bound to the ESE motifs of NFE2L1 exon 4. To test this hypothesis, specific bases within the ESE motifs of exon 4 were mutated, as shown in Fig. 2H. The mutated NFE2L1 minigene plasmids, along with SRSF6-Flag plasmids, were subsequently transfected into 293T cells to assess the impact of these mutations. The results demonstrated a decrease in exon 4 inclusion after mutation of the ESE motifs (Fig. 2I), indicating that SRSF6 directly binds to these ESE motifs to promote exon 4 recognition. From these findings, we can conclude that SRSF6 binds to exon 4 of *NFE2L1* and promotes the expression of *NFE2L1-L* by inhibiting the skipping of exon 4.

### SRSF6 modulates NFE2L1 alternative splicing to regulate ESCC cell proliferation

To assess the expression profile of NFE2L1 in ESCC, we first examined its mRNA levels using data from the TCGA database, which revealed that NFE2L1 was significantly upregulated in tumor tissues (Fig. S3A). Consistent with these findings, analysis of the PRIDE database (PXD021701) demonstrated elevated levels of NFE2L1 protein in both 124 unpaired (Fig. S3B) and paired esophageal cancer patients (Fig. S3C) compared to normal esophageal tissue.

**Figure 3:**
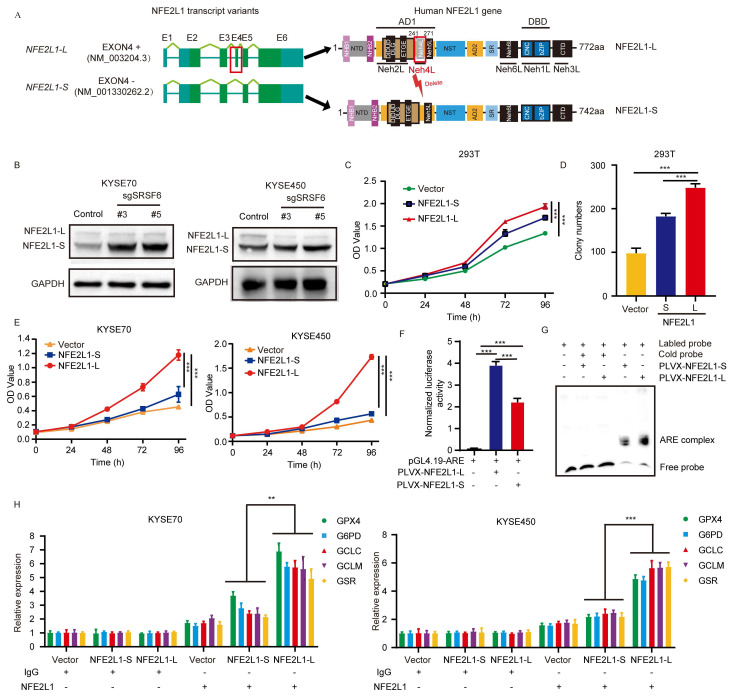
**SRSF6 modulates NFE2L1 alternative splicing to regulate ESCC cell proliferation. A,** Schematic diagram of the long and short isoforms of the human NFE2L1 gene and its encoded protein structure. **B,** Western blot analysis of NFE2L1 isoforms (NFE2L1-L and NFE2L1-S) in KYSE70 and KYSE450 cells transfected with control sgRNA (control) or sgRNAs targeting SRSF6 (#3 and #5). GAPDH was used as a loading control. **C,** The effect of *NFE2L1-L* and *NFE2L1-S* overexpression on 293T cell proliferation was detected by MTT cell proliferation assay. OD values were detected at 0, 24, 48, 72, and 96 h, respectively (n = 3).** D,** The effect of NFE2L1-L and NFE2L1-S overexpression on the anchorage-dependent growth of 293T cells was determined by colony formation assay (n = 3). **E,** Cell viability of KYSE70 and KYSE450 cells expressing vector, NFE2L1-S, or NFE2L1-L was measured at the indicated time points (n = 3). **F,** ARE luciferase reporter activity in 293T cells transfected with pGL4.19-ARE and PLVX-NFE2L1-L or PLVX-NFE2L1-S, as indicated (n = 3). **G,** EMSA using nuclear extracts (5 μg) from 293T cells expressing NFE2L1-L or NFE2L1-S and a biotin-labeled ARE probe, with unlabeled (cold) probe added at 10-fold molar excess for competition. **H,** ChIP-qPCR analysis of NFE2L1 occupancy at the promoter regions of GPX4, G6PD, GCLC, GCLM, and GSR in KYSE70 and KYSE450 cells expressing vector, NFE2L1-S, or NFE2L1-L. Chromatin was immunoprecipitated with anti-NFE2L1 antibody or IgG control, followed by qPCR using primers targeting the indicated promoter regions (n = 3). All the data are presented as the mean ± SD. Data in panels C, D, E, F, H were analyzed by one-way ANOVA. Asterisks indicate statistical significance (***P < 0.001).

The long transcript of human *NFE2L1* comprises six exons 25. Upon analyzing the transcript sequencing data, we found that SRSF6 regulated the skipping of exon 4 in *NFE2L1* to control the transformation between two different isoforms: *NFE2L1-L* (the long isoform) and *NFE2L1-S* (the short isoform) (Fig. 3A). To further validate the isoform-specific alterations in *NFE2L1* expression, we performed Western blot analysis in sgSRSF6 KYSE70 and KYSE450 cells. Consistent with our RT-PCR results, SRSF6 depletion led to a pronounced decrease in the long *NFE2L1* isoform, accompanied by an increase in the short isoform, indicating a shift in alternative splicing (Fig. 3B). Subsequently, we characterized the functional impacts of *NFE2L1* splicing isoforms *NFE2L1-L* and *NFE2L1-S* on 293T cells. Proliferation assays demonstrated that NFE2L1-L enhanced cell proliferation compared to either NFE2L1-S or vector (Fig. 3C). Additionally, clonogenicity was markedly induced in NFE2L1-L expressing cells, as evidenced by increased colony-forming ability (Fig. 3D and Fig. S3D). Consistent with these findings, NFE2L1-L exhibited a more pronounced growth-promoting effect in additional cellular contexts. Specifically, in KYSE70 and KYSE450 cell lines, overexpression of NFE2L1-L further augmented proliferative capacity relative to NFE2L1-S, reinforcing the isoform-specific functional divergence (Fig. 3E). To confirm that the SRSF6 depletion phenotype is mediated through NFE2L1 exon 4 splicing, we performed rescue experiments in SRSF6-depleted ESCC cells, where restoring NFE2L1-L significantly rescued proliferation, while NFE2L1-S had a much weaker effect (Fig. S3E), confirming that SRSF6 regulates tumor growth via exon 4 splicing of NFE2L1.

Previous studies have demonstrated that NFE2L1 partners with small Maf proteins (sMafs) to form a heterodimeric complex that binds to AREs and activates downstream target genes involved in the oxidative stress response 15,18. To investigate the functional differences between NFE2L1-L and NFE2L1-S, we conducted a luciferase reporter gene assay to evaluate transcriptional activity on ARE. Our data revealed that both isoforms exhibit ARE-driven transcriptional activity, with NFE2L1-S displaying significantly reduced activity compared to NFE2L1-L (Fig. 3F). Moreover, we identified the binding ability of NFE2L1 to ARE through an EMSA assay, demonstrating stronger binding of NFE2L1-L than NFE2L1-S (Fig. 3G). Moreover, ChIP-qPCR results demonstrated differential binding of NFE2L1-L and NFE2L1-S to the promoters of GPX4, G6PD, GCLC, glutamate-cysteine ligase modifier subunit (GCLM), and glutathione reductase (GSR) genes in KYSE70 and KYSE450 cells (Fig. 3H). These findings indicate that NFE2L1 isoforms exhibit differential promoter occupancy, suggesting distinct regulatory roles in the expression of antioxidant genes. Taken together, these data demonstrate that SRSF6 regulates ESCC cell growth primarily by modulating NFE2L1 expression and activity through alternative splicing-mediated control of NFE2L1 isoform production.

### Depletion of SRSF6 reduces NFE2L1-regulated antioxidant gene expression, increases ROS, and induces apoptosis and ferroptosis in ESCC cells

**Figure 4:**
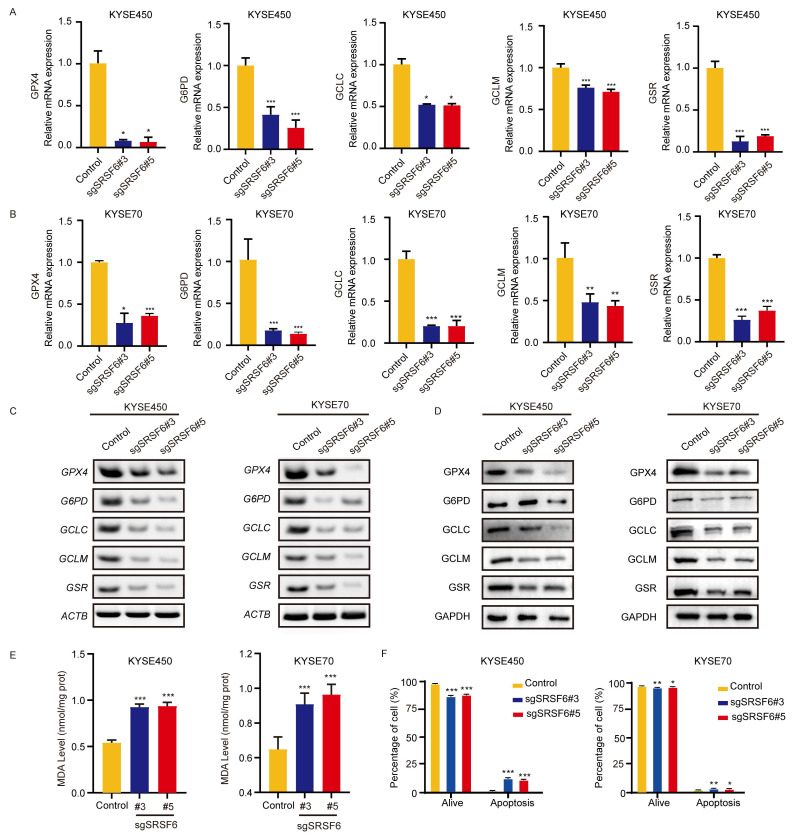
** Depletion of SRSF6 reduces NFE2L1-regulated antioxidant gene expression, increases ROS, and induces apoptosis and ferroptosis in ESCC cells. A, B,** The expressions of GPX4, G6PD, GCLC, GCLM, and GSR in KYSE450 (**A**) and KYSE70 (**B**) cells after SRSF6 knockout were analyzed by qPCR (n = 3). **C,** The expression levels of GPX4, G6PD, GCLC, GCLM, and GSR in KYSE450 and KYSE70 cells were analyzed by RT-PCR following SRSF6 knockout. **D,** The expressions of GPX4, G6PD, GCLC, GCLM, and GSR in KYSE450 and KYSE70 cells after SRSF6 knockout were determined by Western blot.** E,** The lipid peroxidation level of KYSE450 and KYSE70 cells was assessed by MDA quantification after SRSF6 knockout (n = 3). **F,** Apoptotic analysis of KYSE 70 and KYSE450 cells following CRISPR/Cas9-mediated knockout of SRSF6 (n = 3). All the data are presented as the mean ± SD. Data in panels A, B, E, F were analyzed by one-way ANOVA. Asterisks indicate statistical significance (*P < 0.05, **P < 0.01, ***P < 0.001).

RNA sequencing analysis revealed a decrease in the mRNA level of several antioxidant genes containing AREs, including GPX4, G6PD, GCLC, GCLM, and GSR upon SRSF6 knockout. The downregulation of these ARE-containing genes, which are direct transcriptional targets of NFE2L1, is likely to contribute to the impaired antioxidant response 27,28. Subsequently, we conducted qPCR and RT-PCR to observe changes in the mRNA levels of five essential genes after knocking out SRSF6. In line with the results of the qPCR (Fig. 4A and B), the *GPX4*, *G6PD*, *GCLC*, *GCLM*, and *GSR* mRNA levels were significantly reduced in KYSE450 and KYSE70 cells by RT-PCR (Fig. 4C). Western blot results also confirmed the downregulation of protein expression levels for these genes (Fig. 4D).

The upregulation of intracellular ROS can be attributed to the decreased activity of ARE, a crucial cis-acting element involved in the antioxidant process 29. MDA is a product of lipid peroxidation. Excessive ROS levels can lead to depletion of the cellular antioxidant defense system, including GSH, leading to increased lipid peroxidation and, consequently, increased production of MDA 30. The levels of MDA were measured using the thiobarbituric acid reactive substances (TBARS) assay, revealing a significant increase in MDA levels following SRSF6 knockout (Fig. 4E). It is widely recognized that high levels of ROS can trigger apoptosis through both internal and external pathways 31. Our results showed that knocking out SRSF6 induced apoptosis in KYSE450 and KYSE70 cells (Fig. 4F and Fig. S4A). These results suggest that the downregulation of antioxidant genes may disrupt redox homeostasis, leading to excessive oxidative stress. To differentiate the contributions of apoptosis and ferroptosis in the phenotype caused by SRSF6 depletion, we conducted pharmacological rescue experiments in sgSRSF6 ESCC cells using Ferrostatin-1, Deferoxamine, and Z-VAD. As shown in Fig. S4B, all three treatments significantly restored cell viability in both KYSE70 and KYSE450 sgSRSF6 cells compared to the untreated sgSRSF6 group. These results suggest that both apoptosis and ferroptosis contribute to the cell death phenotype induced by SRSF6 depletion. Specifically, partial rescue by Ferrostatin-1 and Deferoxamine supports the involvement of iron-dependent lipid peroxidation, while Z-VAD confirms the role of apoptosis. Thus, SRSF6 depletion induces a mixed cell death phenotype in ESCC cells, with both apoptosis and ferroptosis playing a role in growth inhibition.

### SRSF6 is highly expressed in ESCC, and its expression is regulated by the transcription factor NFE2L1

**Figure 5:**
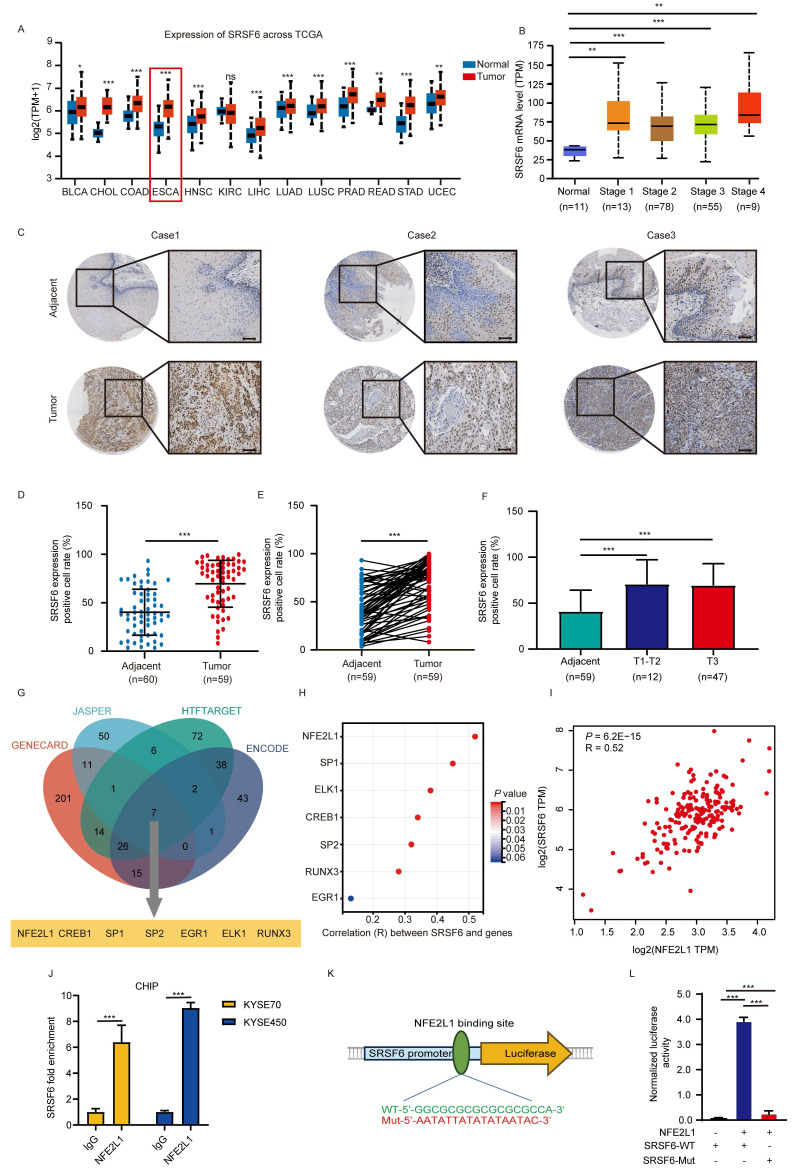
**SRSF6 is highly expressed in ESCC, and its expression is regulated by the transcription factor NFE2L1. A,** Analysis of SRSF6 expression in different cancer types from the TCGA database. **B,** Comparison of SRSF6 mRNA expression in the TCGA database and clinical stage of esophageal cancer. **C,** Representative IHC staining images of ESCC tissue array using SRSF6 antibody in adjacent tissues and paired cancer tissues. Scale bar: 50 μm. **D,** IHC assessed SRSF6 protein levels in ESCC (n = 59) and adjacent (n = 60) tissue samples. **E,** Quantification of SRSF6 expression in paired samples (n = 59). **F,** IHC quantification of SRSF6 positive cell rate in adjacent tissues and tumors stratified by T stage (T1-T2 and T3) (n = 59). **G,** Prediction analysis identified NFE2L1, CREB1, SP1, SP2, EGR1, ELK1, and RUNX3 as potential transcription factors regulating SRSF6 expression in ESCC, based on data from the GeneCards, JASPAR, hTFtarget, and ENCODE databases. **H,** Correlation analysis of SRSF6 with the predicted transcription factors NFE2L1, CREB1, SP1, SP2, EGR1, ELK1, and RUNX3 based on TCGA esophageal cancer data. **I,** Scatter plot illustrating the correlation between SRSF6 and NFE2L1 (R=0.52, *P*=6.2×10^-15^). **J,** ChIP experiments were performed using an NFE2L1 antibody in KYSE70 and KYSE450 cell lines, showing the enrichment levels of the SRSF6 promoter region in the NFE2L1 antibody group compared to the IgG control group (n = 3). **K,** Luciferase reporter constructs containing both wild-type and mutant binding sites were generated. **L,** Co-transfection of NFE2L1 with the SRSF6-WT and SRSF6-Mut constructs displayed variations in luciferase activity across different groups (n = 3). All the data are presented as the mean ± SD. Data in panels A, D, J were analyzed by independent two-sample t-test; panel E by paired t-test; panels B, F, L by one-way ANOVA. Asterisks indicate statistical significance (*P < 0.05, **P < 0.01, ***P < 0.001).

Through analysis of the TCGA database, SRSF6 was found to be highly expressed in multiple tumor types (Fig. 5A), with significant overexpression observed in esophageal cancer among 13 tumor types (*P* = 0.000018). Additionally, increased levels of SRSF6 mRNA were also detected across various clinical stages of ESCC compared to normal esophageal tissues (Fig. 5B). Subsequently, we utilized an ESCC tissue array with patient pathological information to assess SRSF6 protein levels (Fig. 5C). SRSF6 protein was mainly localized in the nucleus, showing a higher positive expression rate in ESCC tissues than in adjacent tissues, indicating that SRSF6 protein levels were elevated in cancerous tissues. Interestingly, analysis of the positive rate of SRSF6 staining revealed that SRSF6 protein levels were significantly increased in unpaired (Fig. 5D) and paired (Fig. 5E) ESCC samples. Further analysis revealed substantially higher SRSF6 protein levels in stages T1, T2, and T3 than in adjacent tissues (Fig. 5F). However, no significant difference in SRSF6 expression was observed among these T stages. We further evaluated whether the expression of SRSF6 was correlated with clinical stage, pathological grade, lymph node metastasis, patient age, and gender in ESCC. However, no significant correlations were observed between SRSF6 expression and age (*P* = 0.942), gender (*P* = 0.711), pathological grade (*P* = 0.087), or lymph node metastasis (*P* = 0.145) (Table 1). To further validate the upregulated expression of SRSF6 in ESCC, we conducted an additional analysis using 124 unpaired esophageal cancer patient tissues from the PRIDE database (PXD021701). Our findings revealed significantly elevated levels of SRSF6 protein expression in ESCC tissues compared to adjacent tissues (Fig. S5A and B) 15. These findings provide compelling evidence for the high expression of SRSF6 in ESCC tissues.

To investigate the reasons for the high expression of SRSF6 in ESCC, we conducted a prediction analysis of potential transcription factors regulating SRSF6. NFE2L1, cAMP response element-binding protein 1 (CREB1), transcription factor Sp1 (SP1), transcription factor Sp2 (SP2), early growth response protein 1 (EGR1), ETS domain-containing protein Elk-1 (ELK1), and runt-related transcription factor 3 (RUNX3) were identified through the intersection of results obtained from the GeneCards, JASPAR, hTFtarget, and ENCODE databases, which were used to predict transcription factors for SRSF6 (Fig. 5G). The correlation between SRSF6 and the predicted transcription factors NFE2L1, CREB1, SP1, SP2, EGR1, ELK1, and RUNX3 was analyzed using TCGA esophageal cancer data, with NFE2L1 showing the strongest correlation with SRSF6 (Fig. 5H). Scatter plot analysis revealed a significant correlation between SRSF6 and NFE2L1 (*P* = 6.2E-15, R = 0.52; Fig. 5I). ChIP experiments were conducted using an NFE2L1 antibody in KYSE70 and KYSE450 cells. The results demonstrated that the SRSF6 promoter was significantly more enriched in the antibody group compared to the IgG control group. This indicates that NFE2L1 binds to the SRSF6 promoter region (Fig. 5J). The binding site of NFE2L1 in the SRSF6 promoter region was validated using a dual-luciferase reporter assay. Luciferase reporter constructs containing both wild-type and mutant binding sites were generated (Fig. 5K). Co-transfection of NFE2L1 with the SRSF6-WT construct resulted in a significant increase in luciferase activity compared to the SRSF6-WT reporter alone (control), whereas co-transfection with the SRSF6-Mut construct showed a significant reduction in luciferase activity compared to the WT group (Fig. 5L). These results indicate that NFE2L1 binds to this promoter region of SRSF6 and promotes its transcription.

### SRSF6 promotes the proliferation of ESCC *in vitro* and* in vivo*

**Figure 6:**
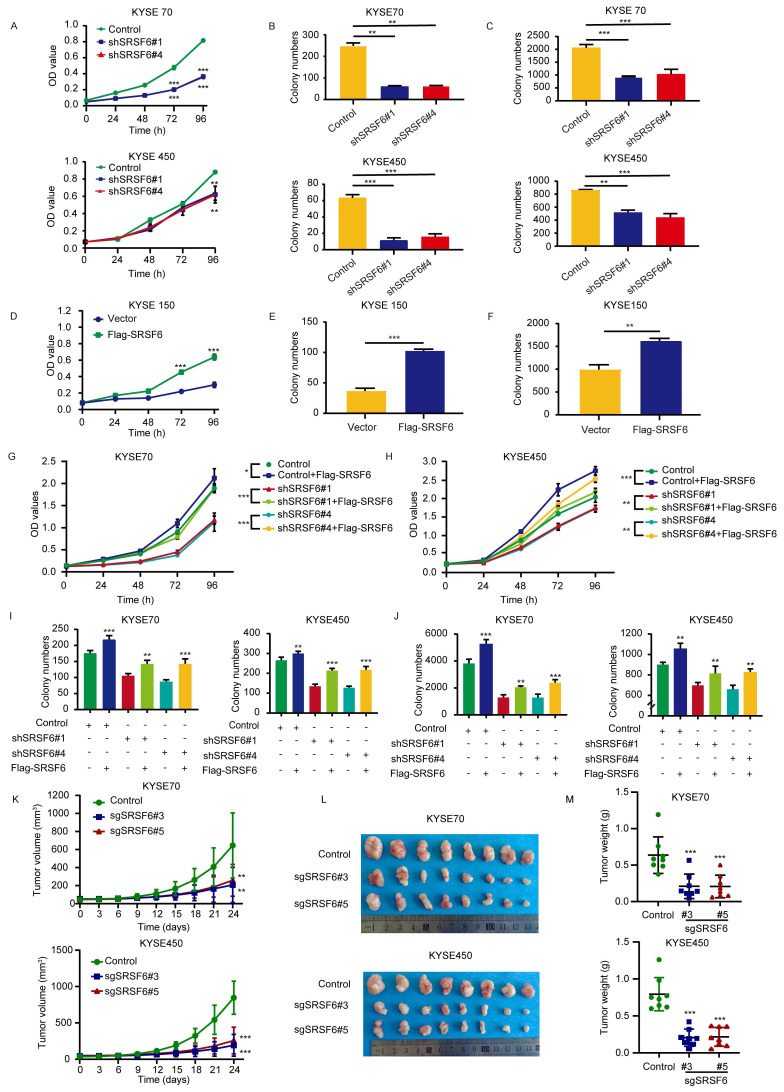
**SRSF6 promotes the proliferation of ESCC *in vitro* and *in vivo*. A,** MTT assay was utilized to ascertain the effect of shRNA-mediated SRSF6 knockdown on the proliferative capacity of KYSE70 and KYSE450 ESCC cell lines. OD values were quantified at 0, 24, 48, 72, and 96 h (n = 3). **B,** The effect of SRSF6 knockdown on the anchor-dependent growth of KYSE70 and KYSE450 cells was determined by plate clone formation assay (n = 3). **C,** The effect of SRSF6 knockdown on the anchorage-independent growth of KYSE70 and KYSE450 cells was determined by soft-agar assay. Colonies were counted for analysis by the IN Cell Analyzer 6000 soft-agar program (n = 3). **D,** The effect of SRSF6 overexpression on KYSE150 cell proliferation was detected by MTT cell proliferation assay. OD values were detected at 0, 24, 48, 72, and 96 h, respectively (n = 3). **E,** The effect of overexpression of SRSF6 on the anchor-dependent growth of KYSE150 cells was determined by plate clone formation assay (n = 3). **F,** The effect of overexpression of SRSF6 on the anchorage-independent growth of KYSE150 cells was determined by soft-agar assay. Colonies were counted for analysis by the IN Cell Analyzer 6000 soft-agar program (n = 3). **G,** Rescue of SRSF6 expression in KYSE70 cells following shRNA-mediated SRSF6 knockdown (shSRSF6#1 or shSRSF6#4) by re-expression of Flag-SRSF6, and cell proliferation was assessed by OD measurements at the indicated time points (n = 3). **H,** Rescue of SRSF6 expression in KYSE450 cells following shRNA-mediated SRSF6 knockdown (shSRSF6#1 or shSRSF6#4) by re-expression of Flag-SRSF6, and cell proliferation was assessed by OD measurements at the indicated time points (n = 3). **I, J,** Colony formation assays in KYSE70 and KYSE450 cells under the indicated conditions (plate colony formation in I; soft agar colony formation in J) (n = 3). **K,** Tumor growth curves after SRSF6 knockout in KYSE70 and KYSE450 cells in a CDX mouse model (n = 8). **L,** Representative tumor images after SRSF6 knockout in CDX mouse models (n = 8). **M,** Gravimetric analysis of knockout SRSF6 tumors in CDX mouse models (n = 8). All the data are presented as the mean ± SD. Data in panels A, B, C, K, M were analyzed by one-way ANOVA, and data in panels D, E, F, G, H, I, J were analyzed by independent two-sample t-test. Asterisks indicate statistical significance (*P < 0.05, **P < 0.01, ***P < 0.001).

Knockdown of SRSF6 in KYSE450 and KYSE70 cells was performed to assess its effect on cell proliferation. Proliferation was reduced by 29.8% and 28% in the shSRSF6#1 and shSRSF6#4 KYSE450 groups, respectively, compared to the control group. Similarly, a reduction of 55.3% and 56.4% was observed in shSRSF6 KYSE70 cells, respectively (Fig. 6A). Furthermore, plate cloning experiments along with soft agar assays demonstrated significant decreases in cloning ability (Fig. 6B and Fig. S6A) as well as anchorage-independent growth capacity among ESCC cells following knockdown of *SRSF6* (Fig. 6C and Fig. S6B).

To further confirm the role of SRSF6 in promoting ESCC cell proliferation, we generated stable SRSF6-overexpressing KYSE150 cells by transfecting pLVX-IRES-puro-3×Flag-SRSF6 plasmids (Fig. S6C). Our MTT assay results (Fig. 6D) demonstrated that overexpression of SRSF6 significantly enhanced anchorage-dependent growth (Fig. 6E and Fig. S6D) and anchorage-independent growth potential (Fig. 6F and Fig. S6E). These findings demonstrated that the overexpression of SRSF6 promotes the proliferation of ESCC cells. Western blot analysis confirmed that re-expression of Flag-SRSF6 successfully restored SRSF6 protein levels in shSRSF6-treated cells (Fig. S6F). In KYSE70 cells, this molecular rescue reduced the shRNA-mediated inhibition of proliferation by 38.06% (Fig. 6G), of clonogenic potential by 26.28% (Fig. 6I and Fig. S6G), and of anchorage-independent growth by 37.06% (Fig. 6J and Fig. S6H). In KYSE450 cells, the corresponding reductions in inhibition were 19.83% for proliferation (Fig. 6H), 37.07% for colony formation (Fig. 6I), and 28.41% for soft-agar growth (Fig. 6J). These results demonstrate that SRSF6 re-expression substantially reversed the suppressive effects of SRSF6 knockdown on ESCC cell proliferation, clonogenicity, and anchorage-independent growth.

To validate the impact of SRSF6 on ESCC cells *in vivo*, we employed sgSRSF6 KYSE450 and KYSE70 cells to create a xenograft model. Our findings revealed that knockout of SRSF6 reduced xenograft tumor growth compared to the control group, as shown by the tumor volume growth curves (Fig. 6K). Representative images of excised tumors are shown in Fig. 6L, and tumor weights were significantly decreased at the endpoint (Fig. 6M). These results unequivocally demonstrated that SRSF6 knockout inhibits the proliferation of ESCC cells *in vivo*. Collectively, our data strongly support the notion that SRSF6 promotes ESCC growth both *in vitro* and* in vivo*.

### ASO-targeting of SRSF6 suppresses ESCC *in vitro* and *in vivo*

**Figure 7:**
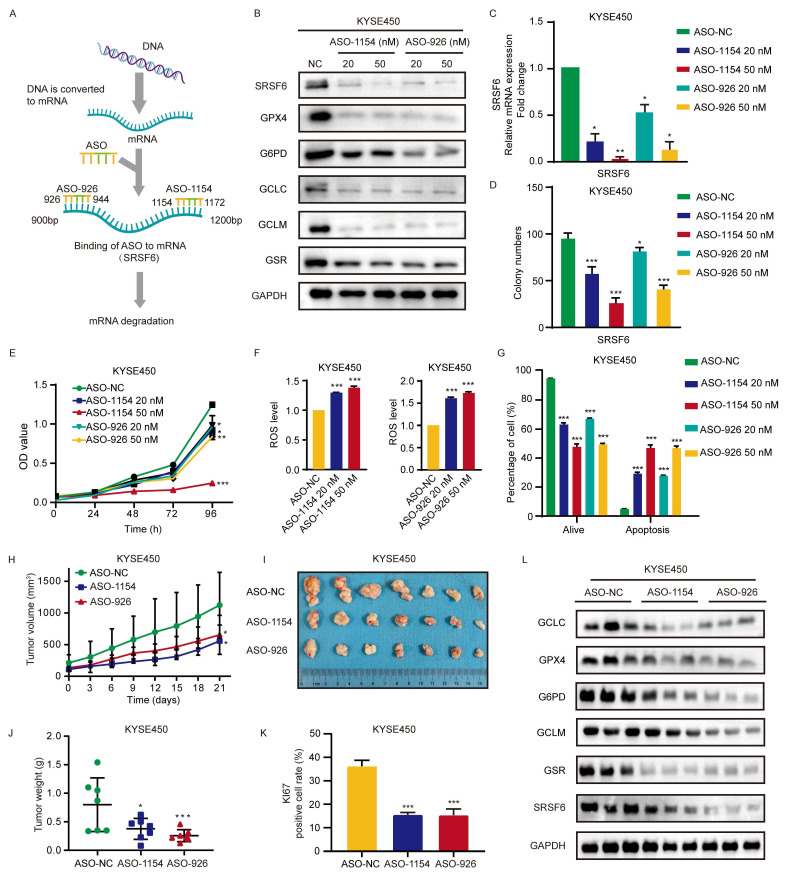
**ASO-targeting of SRSF6 suppresses ESCC *in vitro* and *in vivo*. A,** Mechanism for ASO-induced degradation of SRSF6 in ESCC. **B,** KYSE450 cells were transfected with ASO-NC and ASO-specific targeting sequences ASO-1154 and ASO-926, and a Western blot was performed. **C, D, E,** KYSE450 cells were transfected with ASO-NC and sequence-specific ASO-1154 and ASO-926. RT-qPCR was performed to measure SRSF6 mRNA levels after ASO treatment (C) (n = 3). Clonogenic assays (D) and MTT proliferation assays (E) were performed to evaluate the effects on clonal formation ability and cell proliferation (n = 3). **F,** Intracellular ROS level was examined by DCF staining after transfection with ASO-NC and ASO-specific targets ASO-1154 and ASO-926 in KYSE450 cells (n = 3). **G,** Apoptotic analysis of KYSE450 cells following transfection with ASO-NC and ASO-specific targets ASO-1154 and ASO-926 (n = 3). **H,** The change of average tumor volume in KYSE450 cases after ASO-NC and ASO-specific targets ASO-1154 and ASO-926 treatment (n = 7). **I,** The tumor pictures of the CDX mouse model (n = 7). **J,** The analysis of xenograft tumor weight of the CDX mouse (n = 7). **K,** Statistical analysis of IHC positive staining of Ki67 in KYSE450 cases (n = 3). **L,** Western blot analysis was utilized to examine protein expression levels of the antioxidant genes GPX4, G6PD, GCLC, GCLM, and GSR in CDX tumors. All the data are presented as the mean ± SD. Data for panels C, D, E, F, G, H, J, K were analyzed by one-way ANOVA. Asterisks indicate statistical significance (*P < 0.05, **P < 0.01, ***P < 0.001).

ASO therapy has been proven effective mainly due to its ability to target and degrade mRNA specifically, making it a favored option for cancer treatment 32. In this study, we employed ASO treatment to investigate the possibility of SRSF6 as a therapeutic target due to its high expression and impact on ESCC development. Specifically, designed ASO sequences were utilized to degrade SRSF6 mRNA, followed by treating ESCC cells with 20 nM and 50 nM ASO, respectively (Fig. 7A). The effectiveness of ASO treatment on ESCC cells was checked using Western blot and qPCR analysis. Our results revealed that ASO treatment significantly reduced the protein (Fig. 7B) and mRNA (Fig. 7C) levels of SRSF6. Furthermore, Western blot analysis showed that the protein levels of the antioxidant enzymes GPX4, G6PD, GCLC, GCLM, and GSR were also markedly decreased upon SRSF6-targeting ASO treatment (Fig. 7B). Subsequently, we performed clonogenic assays to assess the effect of ASO-mediated SRSF6 knockdown on ESCC cell proliferative capacity. The clonogenic assay results demonstrated that SRSF6-targeting ASO treatment significantly suppressed the clonogenic growth of ESCC cells compared to ASO-NC treatment (Fig. 7D and Fig. S7A). Cell proliferation assays showed that SRSF6 ASOs decreased cell proliferation in KYSE450 cells (Fig. 7E). Furthermore, the ROS levels in ESCC cells were augmented after ASO-1154 and ASO-926 treatment (Fig. 7F and Fig. S7B). This, in turn, induced apoptosis in KYSE450 cells (Fig. 7G and Fig. S7C). These findings suggest that ASO targeting SRSF6 can inhibit ESCC cell proliferation *in vitro*. In addition, PCR analysis revealed that ASO-1154 and ASO-926 recapitulated the SRSF6-knockdown splicing switch of NFE2L1, markedly decreasing the L isoform and increasing the S isoform (Fig. S7D). This confirms that ASO-mediated targeting of SRSF6 induces NFE2L1 exon 4 skipping, reinforcing the mechanistic link between SRSF6 and NFE2L1 splicing.

Subsequently, we evaluated the effect of ASO treatment targeting SRSF6 on tumor growth *in vivo* utilizing CDX from KYSE450 cells, with high SRSF6 expression in mice. The results showed that compared with ASO-NC, ASO treatment significantly reduced tumor growth as indicated by tumor volume curves (Fig. 7H), yielded smaller excised tumors (Fig. 7I), and decreased tumor weights (Fig. 7J). Moreover, the expression levels of Ki67 also decreased in xenografts after ASO treatment (Fig. 7K and Fig. S7E). The protein levels of SRSF6, GPX4, G6PD, GCLC, GCLM, and GSR, as the downstream pathway of SRSF6, were significantly decreased after ASO treatment (Fig. 7L). These data indicated that ASO treatment targeting SRSF6 had a significant inhibitory effect on ESCC *in vivo*. In summary, ASO targeting SRSF6 suppresses ESCC cell proliferation *in vitro* and *in vivo*, leading to increased ROS levels and thereby restraining the growth of ESCC.

### Enhancement of CDDP sensitivity by targeting SRSF6 in ESCC

**Figure 8:**
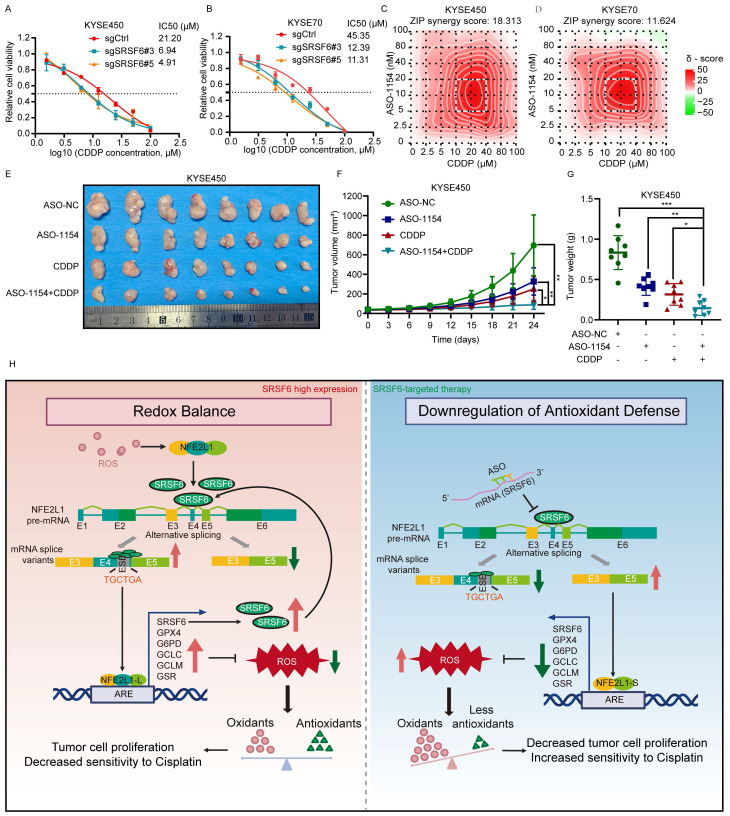
** Enhancement of cisplatin sensitivity by targeting SRSF6 in ESCC. A, B,** Dose-response curves of KYSE450 (A) and KYSE70 (B) cell lines treated with increasing concentrations of CDDP. The IC50 values for each cell line with different CRISPR/Cas9 knockout conditions are shown (n = 3). **C, D,** Synergy score analysis of KYSE450 (C) and KYSE70 (D) cells treated with combinations of ASO-1154 and CDDP. The heatmaps represent the synergy scores (ZIP method) for each drug combination. **E,** Tumor growth in KYSE450 xenograft models treated with ASO-NC, ASO-1154, CDDP, and ASO-1154+CDDP. Representative images of tumors excised at day 24 (n = 8). **F,** Tumor volume progression in KYSE450 xenograft models. Treatment groups include ASO-NC, ASO-1154, CDDP, and ASO-1154 + CDDP (n = 8). **G,** Tumor weight at the endpoint (day 24) (n = 8). **H,** SRSF6 binds to the exonic splicing enhancer in Exon 4 of NFE2L1, preventing exon skipping and promoting the production of full-length NFE2L1 isoforms that support antioxidant defense and ESCC cell proliferation. In turn, NFE2L1 acts as a transcription factor to enhance SRSF6 expression, forming a positive feedback loop. Disruption of this loop, such as by SRSF6-targeting antisense oligonucleotides, increases exon 4 skipping, promotes the generation of the truncated isoform *NFE2L1-S*, elevates intracellular ROS levels, induces apoptosis, and enhances sensitivity to cisplatin, ultimately suppressing ESCC progression. All the data are presented as the mean ± SD. Data for panels F, G were analyzed by one-way ANOVA. Asterisks indicate statistical significance (*P < 0.05, **P < 0.01, ***P < 0.001).

To assess whether targeting SRSF6 can sensitize ESCC cells to CDDP, we first examined the effect of SRSF6 inhibition on cell viability through dose-response assays. The results (Fig. 8A and 8B) demonstrate that SRSF6 knockout significantly reduced the IC50 values for CDDP in both KYSE450 and KYSE70 cells, indicating enhanced CDDP sensitivity. Specifically, in KYSE450 cells, the IC50 values in the SRSF6 knockout group were 6.94 µM and 4.91 µM, compared to 21.20 µM in the control group. Similarly, in KYSE70 cells, the IC50 values were reduced to 12.39 µM and 11.31 µM following SRSF6 knockout, whereas the control group exhibited an IC50 of 45.35 µM. These findings suggest that SRSF6 knockout enhances CDDP sensitivity in ESCC cells by significantly lowering their IC50 values. Next, we evaluated the synergy between ASO-1154 and CDDP using synergy score analysis. The results (Fig. 8C and 8D) revealed significant positive synergy in both cell lines, with synergy scores of 18.313 for KYSE450 and 11.624 for KYSE70, supporting the potential of combining ASO-based therapies with CDDP to enhance its cytotoxic effect. *In vivo*, KYSE450 xenograft models were used to assess the impact of ASO-1154 combined with CDDP on tumor growth. The combination therapy of ASO-1154 and CDDP significantly reduced tumor size and weight compared to control and monotherapy groups (*P* < 0.05), confirming a synergistic antitumor effect (Fig. 8E-G). These results demonstrate that targeting SRSF6 with ASOs enhances CDDP sensitivity *in vitro* and significantly improves its anti-tumor efficacy *in vivo*, providing a promising strategy to increase the therapeutic effectiveness of CDDP in ESCC.

## Discussion

Cancer cells experience elevated oxidative stress and rely on robust redox homeostasis for survival and progression 33. Although RNA splicing has been implicated in redox regulation, the direct involvement of splicing factors and their functional interplay with transcriptional control in oxidative stress adaptation remain poorly understood. SRSF6 is a key oncogenic splicing factor that modulates alternative splicing of tumor-associated genes in multiple cancers, including basal cell carcinoma, squamous cell carcinoma, and melanoma 17. In colorectal cancer, SRSF6 promotes tumor progression by modulating ZO-1 splicing through direct binding to exon 23 12. Additionally, SRSF6 binding to alternative tenascin C exons promotes isoforms associated with invasive and metastatic cancer 17. Elevated SRSF6 also correlates with poor prognosis in T-cell acute lymphoblastic leukemia 34. These findings show that SRSF6 regulates alternative splicing of key genes involved in tumor progression and metastasis. Here, our study indicates that SRSF6 is highly expressed in ESCC and is positively correlated with a poor prognosis. Although SRSF6 expression did not correlate significantly with clinicopathologic variables (Table 1), its prognostic impact likely reflects its role in modulating redox homeostasis and therapy sensitivity, which can influence survival independently of conventional stage or grade. Mechanistically, SRSF6 and NFE2L1 form a positive feedback loop that maintains redox balance in ESCC cells: NFE2L1 functions as a transcription factor that promotes SRSF6 expression, while SRSF6 prevents skipping of NFE2L1 exon 4, ensuring proper expression of the long isoform that supports antioxidant defense. This reciprocal regulation directly links RNA splicing to oxidative stress control, allowing ESCC cells to sustain redox homeostasis and enhance tumor progression (Fig. 8H). Our findings uncover the SRSF6-NFE2L1 regulatory axis as a critical determinant of redox balance and tumor progression, establishing SRSF6 as a distinctive therapeutic target and offering a promising strategy for ESCC treatment.

As a member of the SR protein family, SRSF6 recognizes ESEs and regulates the alternative splicing of genes involved in cancer 12,13,17,35. Our study identifies a previously unrecognized mechanism: SRSF6 directly controls alternative splicing of NFE2L1 exon 4 in ESCC. Using an ESE finder tool (http://exon.cshl.edu/ESE/), we identified putative ESE sites flanking exon 4 that may interact with SRSF6. Our findings, supported by RIP assays, minigene reporter assays, and ESE motif mutagenesis, confirmed that SRSF6 binding inhibits exon 4 skipping, resulting in a preference for the NFE2L1-L isoform. This regulation has functional implications, as NFE2L1-L exhibits higher transcriptional activity on ARE than its shorter counterpart, thereby enhancing the antioxidant capacity of ESCC cells. Moreover, the elevated expression of the NFE2L1-L isoform observed in ESCC further underscores its potential role in supporting tumor cell survival under oxidative stress. Due to impaired *NFE2L1* splicing, the expression of antioxidant enzymes such as GPX4, G6PD, GCLC, GCLM, and GSR decreases at both the mRNA and protein levels, resulting in elevated ROS and lipid peroxidation. GPX4 utilizes the primary cellular antioxidant GSH to reduce lipid peroxides, generating oxidized GSSG, which is recycled back to GSH by GSR in an NADPH-dependent process 36,37. G6PD-produced NADPH is central to redox homeostasis, acting as a cofactor for glutathione reductase in oxidized glutathione recycling 38. GCLC and GCLM are responsible for de novo GSH synthesis and play pivotal roles in detoxification, antioxidant defense, and maintenance of thiol status 39-41. These enzymes contain AREs in their promoter regions that are activated by NFE2L1 42. Collectively, these findings highlight SRSF6-mediated NFE2L1 splicing as a critical mechanism for maintaining redox balance in ESCC cells.

Redox homeostasis, a precise balance between oxidants and antioxidants, is essential for normal cellular function; its disruption causes oxidative stress, leading to aberrant ROS signaling, DNA mutations, and genomic instability, which are key drivers of cancer development 43,44. Therefore, elevating ROS levels or targeting ROS scavenging pathways may selectively induce tumor cell death 45. The increased antioxidant status in tumor cells optimizes ROS-driven proliferation while evading apoptosis 46. To counteract the ROS generated by aberrant metabolism, tumor cells upregulate transcription factors associated with antioxidant enzymes, such as NFE2L1 and NFE2L2 6,47. In this context, our discovery of the SRSF6-NFE2L1 axis adds a previously unrecognized layer of post-transcriptional control to this adaptive program. The crucial importance of this axis for ESCC lies in its ability to simultaneously maintain redox homeostasis and limit ferroptosis. By ensuring robust expression of the long NFE2L1 isoform, SRSF6 sustains the transcription of GPX4 and GSH-related enzymes, thus suppressing lipid peroxidation-driven ferroptosis-a cell death mode that is intimately linked to therapy resistance in ESCC. This likely explains why SRSF6 depletion not only impairs antioxidant defense but also triggers ROS-dependent apoptosis and ferroptosis, rendering cancer cells more vulnerable. Moreover, NFE2L1 itself promotes SRSF6 transcription, establishing a self-reinforcing positive feedback loop that locks cancer cells into an oxidation-resistant state. This regulatory circuitry may contribute to ESCC progression: the feedback loop provides a sustained fitness advantage under the high oxidative burden of the tumor microenvironment and may contribute to chemoresistance, as commonly used agents such as cisplatin rely in part on ROS-mediated cytotoxicity 48. Accordingly, the prognostic impact of SRSF6 can be understood through this functional link to redox and ferroptosis regulation.

The significance of the SRSF6-NFE2L1 axis may extend well beyond ESCC. SRSF6 is overexpressed in numerous malignancies, including lung adenocarcinoma, melanoma, and colorectal cancer, and aberrant NFE2L1 splicing or altered antioxidant capacity is a common feature of these tumors 27,35. In light of our findings, it is plausible that SRSF6-driven NFE2L1 isoform switching represents a general mechanism by which cancer cells adapt to oxidative stress and evade ferroptosis. Targeting this axis could therefore offer a broad therapeutic strategy, either by directly inhibiting SRSF6 to disrupt the redox feedback loop or by inducing ferroptosis through pharmacologically impairing the NFE2L1-dependent antioxidant program. Future studies exploring SRSF6-NFE2L1 dependency across different tumor types will be valuable to evaluate its potential as a pancancer vulnerability.

Targeting abnormal splicing shows potential for treating cancer, as splicing factors become appealing therapeutic targets 49. Trans-acting splicing regulatory proteins, including SR and hnRNP proteins, impact cancer cell death pathways 50. Clinical trials are testing splicing factor inhibitors, but no specific inhibitors for SRSF6 51. Previous studies have demonstrated that ASOs can effectively correct aberrant splicing and achieve therapeutic benefits against splicing factor targets in cancer models. For example, converting aberrant in SF3B1-mutant uveal melanoma cells using ASOs restores Bromodomain-containing protein 9 levels and demonstrates therapeutic effects *in vitro* and *in vivo* 51. Our finding that targeting SRSF6 with an ASO sensitizes ESCC cells to cisplatin aligns with this paradigm, yet is distinct in its underlying mechanism: SRSF6 inhibition disrupts the SRSF6-NFE2L1 positive feedback loop, impairs the antioxidant defense network, and elevates ROS, thereby enhancing cisplatin cytotoxicity. This highlights a principle by which splicing factor-directed ASOs can be leveraged to resensitize tumors to conventional chemotherapy. In a broader clinical context, combining ASO-based splicing inhibition with cisplatin may offer a strategy to overcome chemoresistance not only in ESCC but also in other malignancies where redox-active splicing programs sustain tumor survival.

SRSF6-targeted ASO therapy sensitized ESCC cells to cisplatin in our study. However, several translational limitations must be considered before clinical application. Notably, the intratumoral delivery employed here differs substantially from systemic administration, and does not fully recapitulate the pharmacokinetics, biodistribution, or off-target exposure expected in patients. Furthermore, our study did not include systemic tolerability or toxicity readouts, which are essential for evaluating the therapeutic window of an SRSF6-targeted ASO. Clinical experience with systemically administered ASOs has revealed class-specific toxicities, including hepatotoxicity, nephrotoxicity, thrombocytopenia, and pro-inflammatory effects through TLR activation 52. For instance, the ASO inotersen carries a black-box warning for severe thrombocytopenia, and hepatotoxicity has been a recurrent concern across multiple ASO platforms 53. Therefore, future translation of SRSF6-targeted ASOs will require careful optimization of delivery, comprehensive toxicological profiling, and the establishment of a safe therapeutic index to minimize these risks.

## Conclusions

This study demonstrates that SRSF6 plays a critical oncogenic role in ESCC by regulating redox homeostasis through a previously unrecognized SRSF6-NFE2L1 regulatory axis. SRSF6 promotes tumor progression by controlling alternative splicing of NFE2L1, thereby sustaining antioxidant gene expression, limiting oxidative stress, and enhancing tumor cell survival. Importantly, disruption of SRSF6-mediated splicing impairs redox balance, increases ROS accumulation, and sensitizes ESCC cells to CDDP treatment. These findings not only provide mechanistic insight into the functional interplay between RNA splicing and oxidative stress regulation in cancer but also highlight SRSF6 as a promising therapeutic target. Targeting SRSF6, particularly through ASO-based strategies, may represent a novel and clinically relevant approach to improve treatment outcomes for patients with ESCC.

## Supplementary Material

Supplementary figures and tables.

## Figures and Tables

**Table 1:** The correlation between SRSF6 expression level and clinicopathologic indexes in ESCC patients.

Clinicopathological characteristics		Number (n=59)	SRSF6		*P*
			Low (n=30)	High (n=29)	
Age	<60	14	7 (23.3%)	7 (24.1%)	0.942
	>60	45	23 (76.7%)	22 (75.9%)	
Gender	male	42	22 (73.3%)	20 (69.0%)	0.711
	female	17	8 (26.7%)	9 (31.0%)	
Pathology grade	I -II	32	13 (43.3%)	19 (65.5%)	0.087
	III	27	17 (56.7%)	10 (34.5%)	
T classification	T1-T2	12	4 (13.3%)	8 (27.6%)	
	T3	47	26 (86.7%)	21 (72.4%)	0.174
Lymph node status	N0	33	14 (46.7%)	19 (65.5%)	
	N1-N3	26	16 (53.3%)	10 (34.5%)	0.145

## Data Availability

All original proteomics and phosphoproteomics data of this study were deposited to iProX (ProteomeXchange ID: PXD035562). All original RNA-Seq data generated in this study have been deposited in the NCBI Sequence Read Archive (SRA) under accession number PRJNA861875. The data supporting this study's findings are available from the corresponding author upon reasonable request.

## Abbreviations

A3SS: Alternative 3′ splice site
A5SS: Alternative 5′ splice site
ARE: Antioxidant response element
AS: Alternative splicing
ASO: Antisense oligonucleotide
CDDP: Cisplatin
CDX: Cell-derived xenograft
ChIP: Chromatin immunoprecipitation
ChIP-qPCR: Chromatin immunoprecipitation followed by quantitative PCR
CREB1: cAMP response element-binding protein 1
DCF: 2′,7′-dichlorofluorescein
DCFH-DA: 2′,7′-Dichlorodihydrofluorescein diacetate
EGR1: Early growth response protein 1
ELK1: ETS-like transcription factor Elk-1
EMSA: Electrophoretic mobility shift assay
ESCC: Esophageal squamous cell carcinoma
ESE: Exonic splicing enhancer
G6PD: Glucose-6-phosphate dehydrogenase
GAPDH: Glyceraldehyde-3-phosphate dehydrogenase
GCLC: Glutamate-cysteine ligase catalytic subunit
GCLM: Glutamate-cysteine ligase modifier subunit
GO: Gene Ontology
GPX4: Glutathione peroxidase 4
GSEA: Gene Set Enrichment Analysis
GSH: Reduced glutathione
GSR: Glutathione reductase
GSSG: Oxidized glutathione
IHC: Immunohistochemistry
KEGG: Kyoto Encyclopedia of Genes and Genomes
MDA: Malondialdehyde
MSigDB: Molecular Signatures Database
MTT: 3-(4,5-dimethylthiazol-2-yl)-2,5-diphenyltetrazolium bromide
MXE: Mutually exclusive exons
NAB1: NGFI-A-binding protein 1
NFE2L1: Nuclear factor erythroid 2-related factor 1
NFE2L2: Nuclear factor erythroid 2-related factor 2
PRIDE: PRoteomics IDEntifications database
qPCR: Quantitative (real-time) PCR
RI: Retained intron
RIP: RNA-binding protein immunoprecipitation
RIPA: Radioimmunoprecipitation assay (lysis buffer)
rMATS: Replicate Multivariate Analysis of Transcript Splicing
ROS: Reactive oxygen species
RUNX3: Runt-related transcription factor 3
SDS-PAGE: Sodium dodecyl sulfate-polyacrylamide gel electrophoresis
SE: Skipped exon
sgRNA: Single-guide RNA
shRNA: Short hairpin RNA
SHEE: Immortalized human esophageal epithelial cell line
SMARCA1: SWI/SNF-related, matrix-associated, actin-dependent regulator of chromatin, subfamily A, member 1
SOD: Superoxide dismutase
SRSF6: Serine/arginine-rich splicing factor 6
TBARS: Thiobarbituric acid reactive substances
