# Supplementary material for: Targeting SRSF6 to Enhance Cisplatin Sensitivity by Modulating Redox Balance via NFE2L1 exon 4 Splicing in ESCC
Source: Int J Biol Sci. 2026 Jun 10;22(11):6237–60. doi: 10.7150/ijbs.131590 (PMC13282889; doi:10.7150/ijbs.131590)
Supplement: Supplementary file 1 — Supplementary figures and tables. [file ijbsv22p6237s1.pdf]

Supplemental Figures and Figure Legends

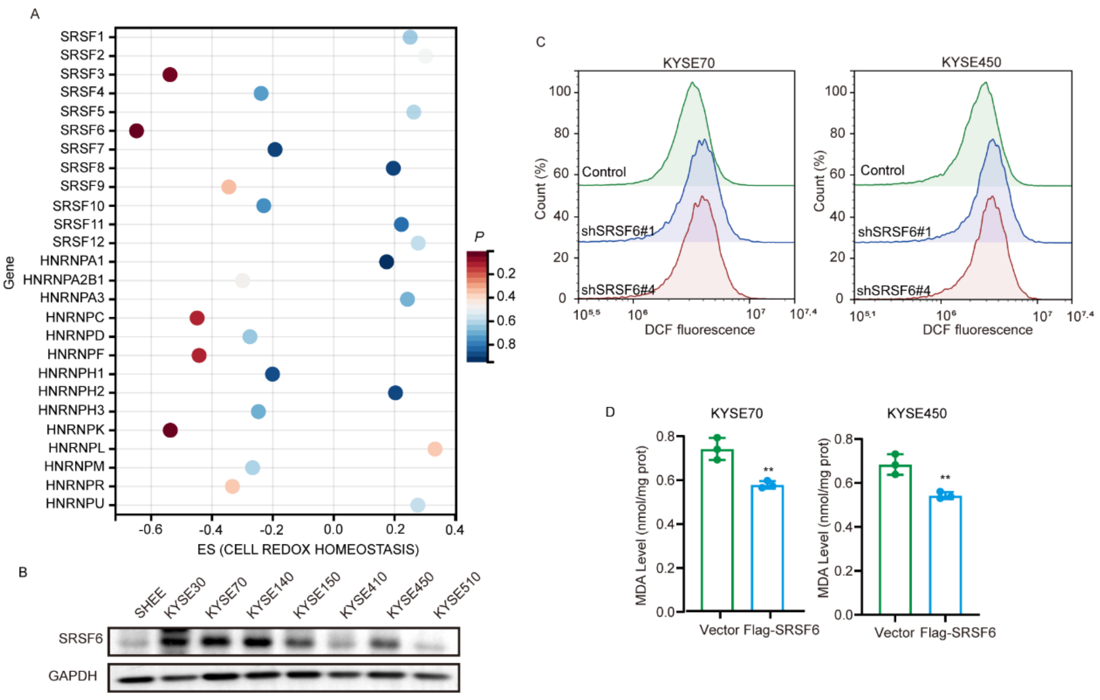

Figure S1 Splicing regulatory proteins influence cell redox homeostasis.

A, A correlation plot of various splicing regulatory proteins and their corresponding enrichment score (ES) values obtained from Gene Set Enrichment Analysis (GSEA), with the x-axis representing the ES scores. Each point represents a splicing regulatory protein, and color intensity reflects the statistical significance.

B, SRSF6 protein levels in normal esophageal immortalized and ESCC cell lines.

C, Intracellular ROS level was examined by DCF staining, after SRSF6 knockout in KYSE70 and KYSE450 cells.

D, Measurement of MDA levels in KYSE70 and KYSE450 cells transfected with control or Flag-SRSF6. Data are presented as mean ± SD (n = 3). Asterisks indicate statistical significance (**$P < 0.01$).

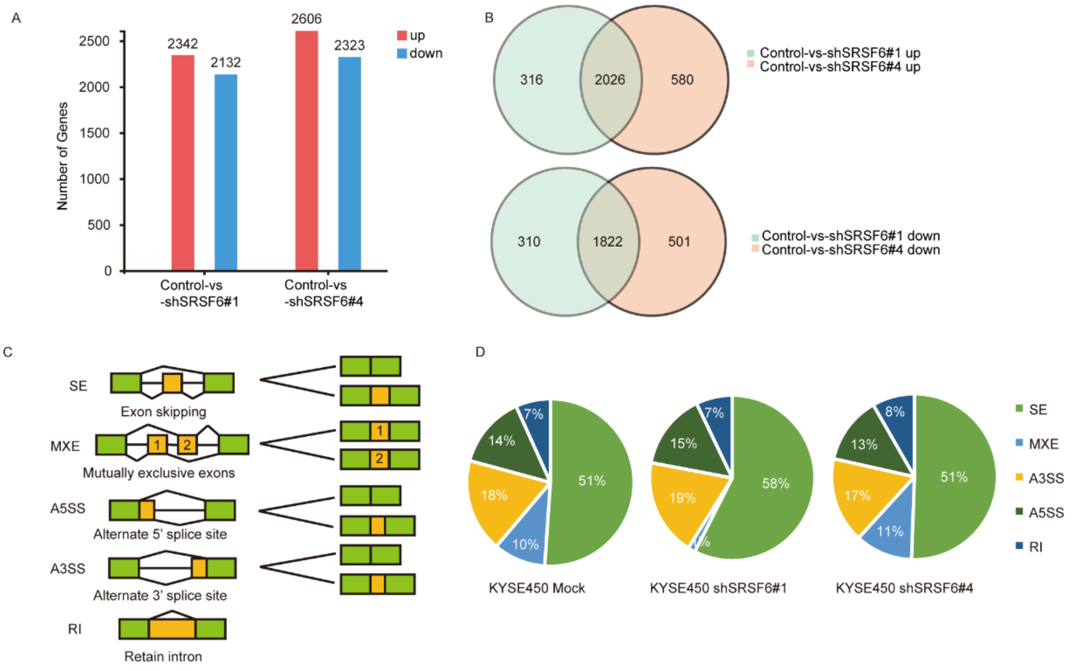

Figure S2 Knocking down SRSF6 promoted NFE2L1S isoform.

A, The number of genes down-regulated in the SRSF6 knockdown group was shown by the Wayne diagram compared with the control group.

B, The Venn diagram showed the number of up-regulated genes in the SRSF6 knockdown group compared to the control group.

C, Schematic diagram of different types of variable splicing: exon jump (SE), exon mutual exclusion (MXE), end site variable splicing, and intron retention.

D, The pie chart shows the percentage of five types of splicing events in KYSE450 cells with SRSF6 down-tapping detected by transcriptional sequencing.

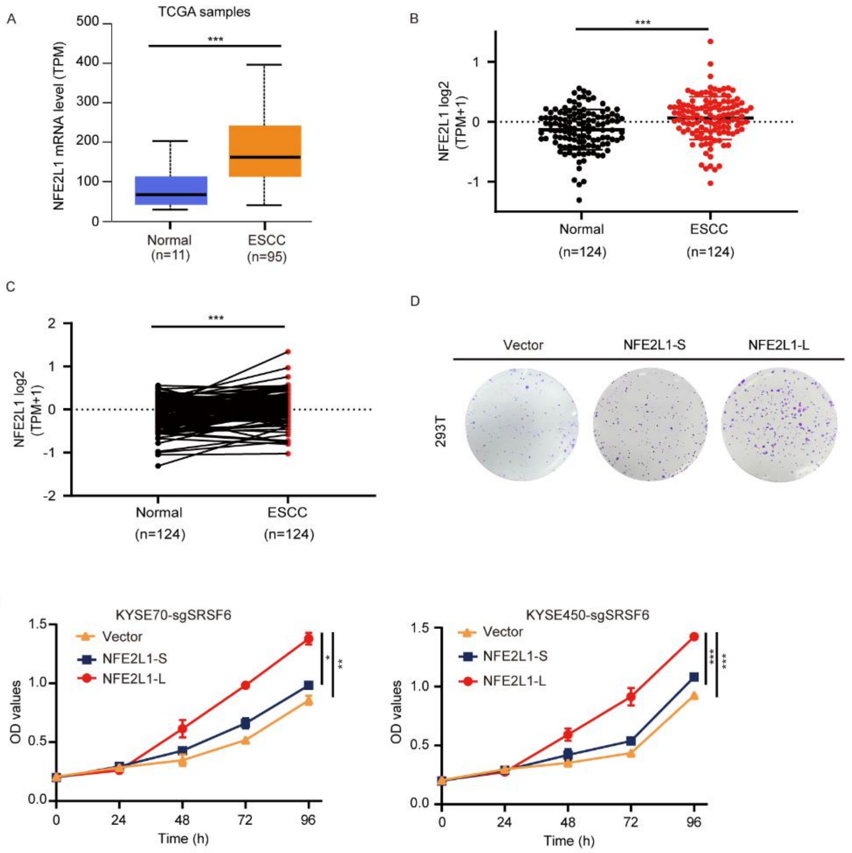

Figure S3 NFE2L1 is highly expressed in tumors.

A, The mRNA expression levels of NFE2L1 in the tumor.

B-C, Phosphorylomic data showed the expression of NFE2L1 in tumor tissues and both unmatched (B) and matched (C) adjacent normal tissues obtained from a cohort of 124 patients with ESCC.

D, Representative images of NFE2L1L or NDE2L1S overexpression in 293T cell lines in the plate clone formation assay.

E, Cell proliferation of KYSE70-sgSRSF6 and KYSE450-sgSRSF6 cells transfected with Vector, NFE2L1-S, or NFE2L1-L was measured at indicated time points using OD values. Data are presented as mean ± SD (n = 3). Asterisks indicate statistical significance (*$P < 0.05$, **$P < 0.01$, ***$P < 0.001$).

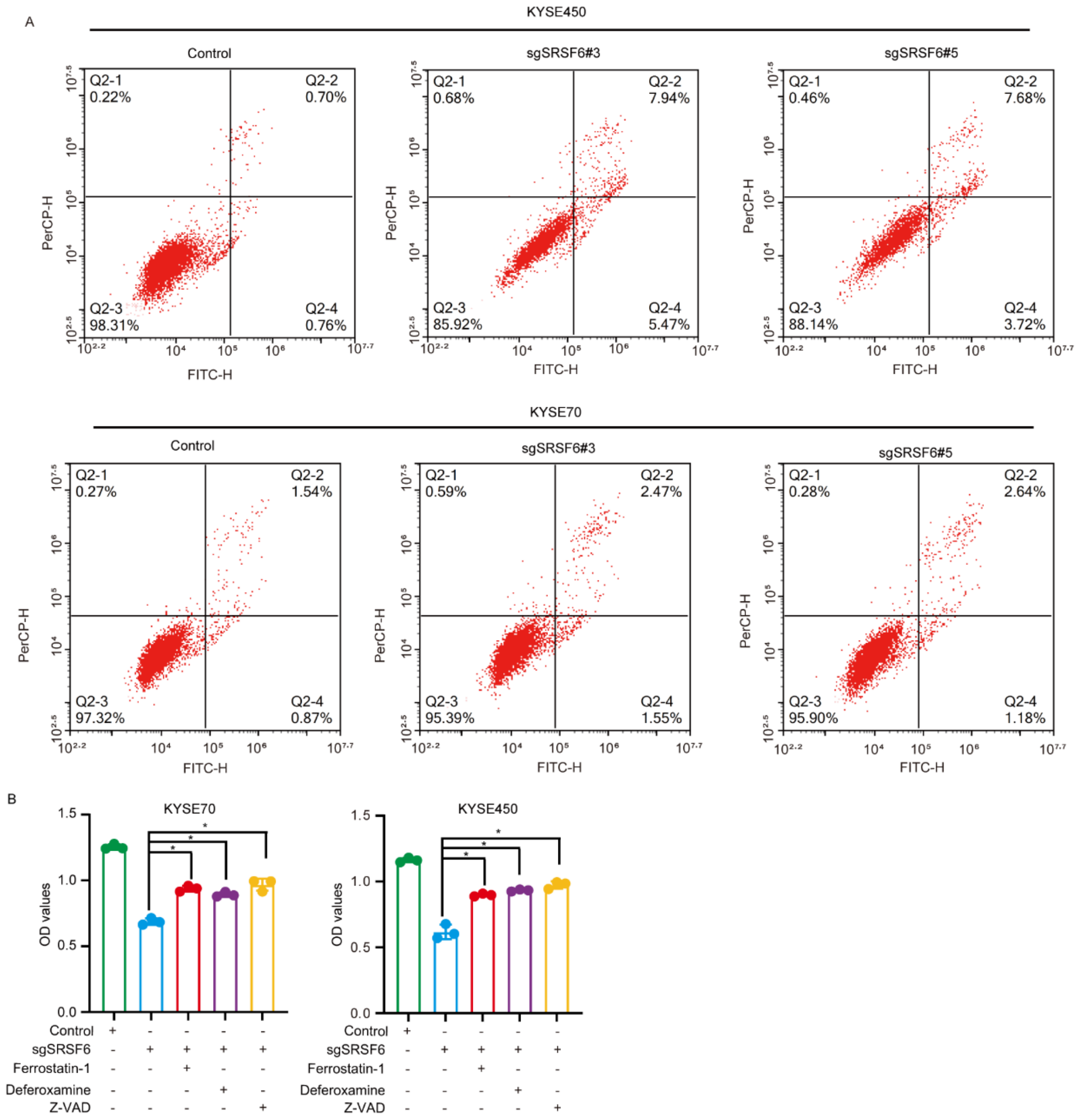

Figure S4 Knocking out SRSF6 induces apoptosis and ferroptosis.

A, Representative flow cytometry histograms depicting apoptosis in KYSE450 and KYSE70 cell lines following CRISPR/Cas9-mediated knockout of SRSF6.

B, Representative flow cytometry histograms depicting apoptosis in KYSE450 and KYSE70 cell lines following CRISPR/Cas9-mediated knockout of SRSF6. Cell viability of KYSE70 and KYSE450 cells transfected with control or sgSRSF6 and treated with Ferrostatin-1 (1 μM), Deferoxamine (100 μM), or Z-VAD (50 μM). OD values were measured at 96 hours post-treatment. Data are presented as mean ± SD (n

= 3). Asterisks indicate statistical significance (*P < 0.05).

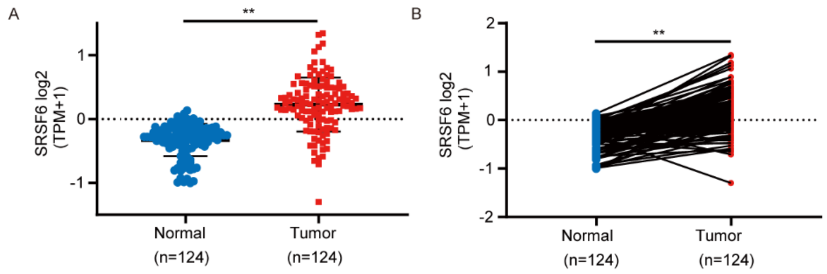

Figure S5 SRSF6 was highly expressed in ESCC.

A-B, Phosphorylomic data showed the expression of SRSF6 in 124 unpaired (A) or paired (B) esophageal carcinoma and normal esophageal tissues. Asterisks indicate statistical significance (**P < 0.01).

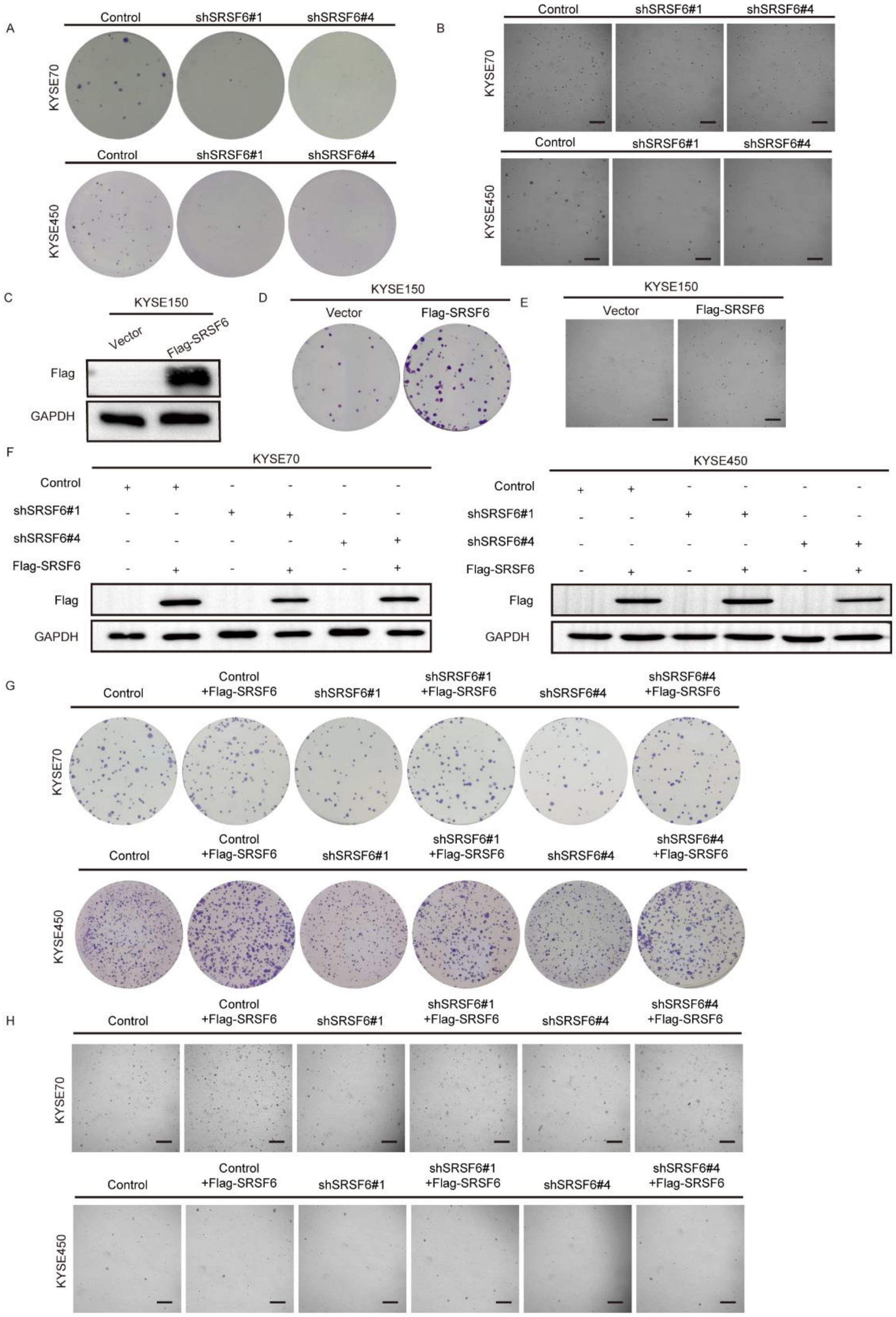

Figure S6 SRSF6 promotes ESCC proliferation.

A-B, Representative images of KYSE70 and KYSE450 SRSF6 knockdown cell lines in plate clone formation assay (A) and anchorage-independent growth assay (B). Representative images of KYSE70 and KYSE450 SRSF6 knockdown cell lines in

anchorage-independent growth assay.

C, The protein levels of SRSF6 protein in KYSE150 cells with SRSF6 overexpression were determined by Western blot assay.

D-E, Representative images of KYSE150 SRSF6 overexpression cell lines in plate clone formation assay (D) and anchorage-independent growth assay (E).

F, The expression level of SRSF6 was rescued in SRSF6 knockdown cell lines KYSE70 and KYSE450 by Western blotting.

G-H, Representative images of anchorage-dependent growth (G) and anchorage-independent growth (H) in SRSF6 recovered shSRSF6 KYSE70 and KYSE450 cells.

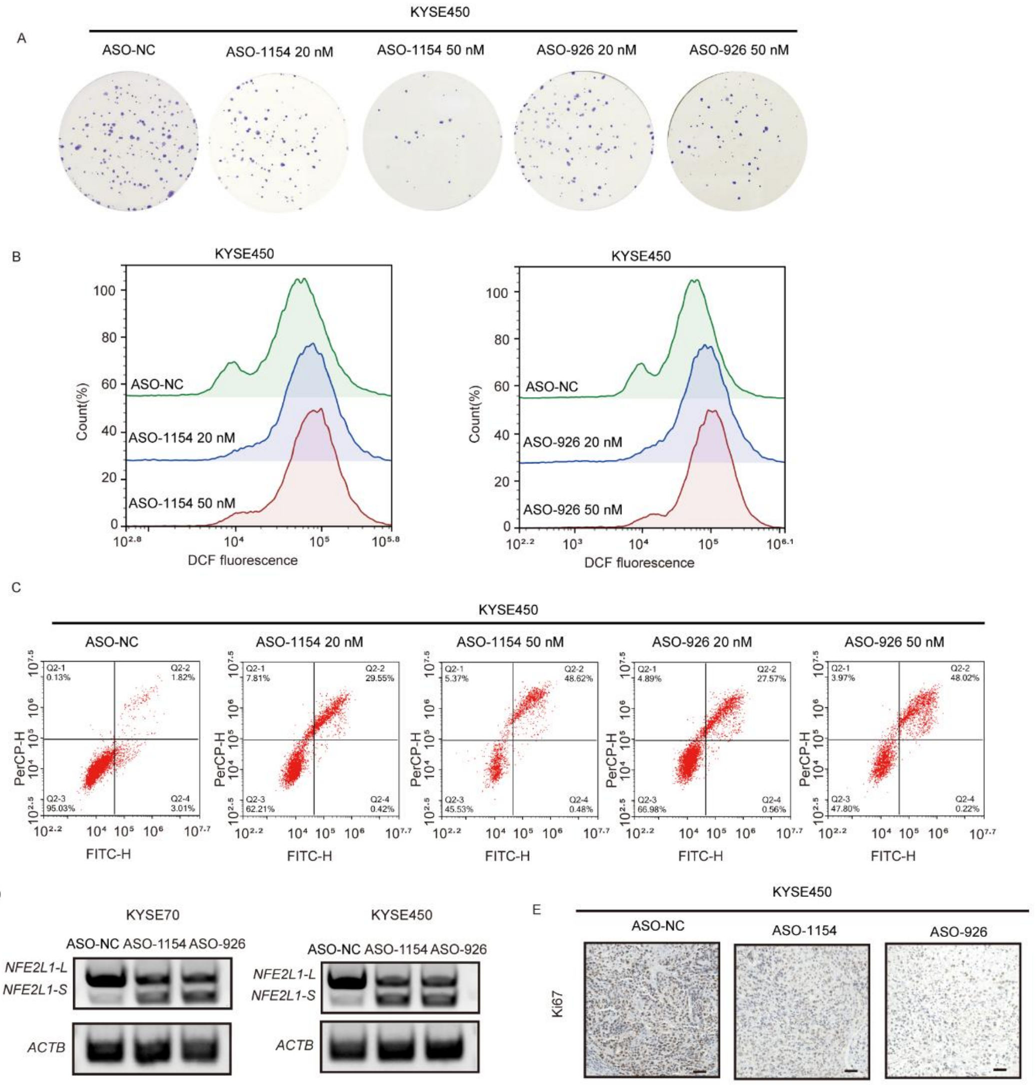

Figure S7 ASOs inhibit the proliferation of ESCC.

A, The representative colony images of KYSE450 after ASO (ASO-NC) and ASO-specific targets ASO-1154 and ASO-926 treatment.

B, The representative images of intracellular ROS level, examined by DCF staining, after being transfected with negative control ASO (ASO-NC) and ASO-specific targets ASO-1154 and ASO-926 in KYSE450 cells.

C, Representative flow cytometry histograms depicting apoptosis after transfection with negative control ASO (ASO-NC) and ASO-specific targets ASO-1154 and ASO-

926 in KYSE450 cells.

D, Representative PCR gel showing the amplification of *NFE2L1-L* and *NFE2L1-S* isoforms in KYSE70 and KYSE450 cells treated with ASO-NC, ASO-1154, or ASO-926. *ACTB* was used as a loading control.

E, Representative IHC images of tumor tissue slices, tumor tissues were stained with Ki67.

Table S1. Antibodies list.

Antibodies	SOURCE	IDENTIFIER
SRSF6	Santa Cruz Biotechnology	sc-57954
SRSF6	Thermo Fisher Scientific	A303-669A
NFE2L1	Santa Cruz Biotechnology	sc-515360
NFE2L1	Proteintech	12936-1-AP
G6PD	Santa Cruz Biotechnology	sc-373886
GCLC	Santa Cruz Biotechnology	sc-166356
GCLM	Santa Cruz Biotechnology	sc-55586
GPX4	Abcam	ab125066
GSR	Santa Cruz Biotechnology	sc-133245
GAPDH	Proteintech	60004-1-Ig
Ki67	Abcam	ab15580

Table S2. The oligonucleotide sequences of SRSF6 shRNA.

Gene Name	Primer sequences 5'-3'
shSRSF6#1-F	CCGGCGTACAGAATACAGGCTTATTCTCGAGAATA AGCCTGTATTCTGTACGTTTTTG
shSRSF6#1-R	AATTCAAAAACGTACAGAATACAGGCTTATTCTCG AGAATAAGCCTGTATTCTGTACG
shSRSF6#4-F	CCGGCGAACAAATGAGGGTGTAATTCTCGAGAAT TACACCCTCATTTGTTCGTTTTTG
shSRSF6#4-R	AATTCAAAAACGAACAAATGAGGGTGTAATTCTCG AGAATTACACCCTCATTTGTTCG

F = Forward primer, R = Reverse primer.

Table S3. The oligonucleotide sequences of SRSF6 single guide (sg) RNA.

Gene Name	Primer sequences 5'-3'
sgSRSF6#3-F	CACCCGACGCCGACGACGCCGTTT
sgSRSF6#3-R	AAACAAACGGCGTCGTCGGCGTCG
sgSRSF6#5-F	CACCCGTCGCGATCGCGACGGCTA
sgSRSF6#5-R	AAACTAGCCGTCGCGATCGCGACG

F = Forward primer, R = Reverse primer.

Table S4. Primer sequences used for qPCR.

Gene Name	Primer sequences 5'-3'
GPX4-F	ACAAGAACGGCTGCGTGGTGAA
GPX4-R	GCCACACACTTGTGGAGCTAGA
GCLM-F	TCTTGCCTCCTGCTGTGTGATG
GCLM-R	TTGGAAACTTGCTTCAGAAAGCAG
GCLC-F	GGAAGTGGATGTGGACACCAGA
GCLC-R	GCTTGTAGTC AGGATGGTTTGCG
G6PD-F	CTGTTCCGTGAGGACCAGATCT
G6PD-R	TGAAGGTGAGGATAACAGGC
GSR-F	TATGTGAGCCGCCTGAATGCCA
GSR-R	CACTGACCTCTATTGTGGGCTTG
ACTB-F	CACCATTGGCAATGAGCGGTTC
ACTB-R	AGGTCTTTGCGGATGTCCACGT

F = Forward primer, R = Reverse primer.

Table S5. Primer sequences used for RT-PCR.

Gene Name	Primer sequences 5'-3'
SRSF6-F	GTGCTTTGGACAAACTGGATGGC
SRSF6-R	CTCCTACTTCGTGACCGTCTTC
NFE2L1-F	ATCTGATTGACATCCTTTGGC
NFE2L1-R	CTGCATTTCCATGATGGACAT
SMARCA1-F	GTTCAAGGGTTCTCATTTTCAGC
SMARCA1-R	CATAGCTTGTAGATCAACCTGTG
RNF138-F	TCGCCATCGCCTTGTTTC
RNF138-R	ATTCTCTTGGTAAGTTTCTGTGTT
NAB1-F	GTCAAAATGTGGAGAAAGAGA
NAB1-R	CTTGTCCATCTGAGTTATCGG
IRF3-F	GAAGACATTCTGGATGAGTTACTG
IRF3-R	CTTGACCATCACGAGCCT
NFE2L1 minigene-F	ATCTGATTGACATCCTTTGGC
NFE2L1 minigene-R	CTGCATTTCCATGATGGACAT
NFE2L1 mutation minigene-F	TCCTGTTGCCACAGGTGTCAGTGGGGAGGACCAGA
NFE2L1 mutation minigene-R	TCTGGTCCTCCCCACTGAGCACCTGTGGCAACAGGA

RIP NFE2L1 Primer1-F	ATCTGATTGACATCCTTTGGC
RIP NFE2L1 Primer1-R	CTGTGCAGGGAAGCTCTC
RIP NFE2L1 Primer2-F	GTGCCTAGTGGGGAGGAC
RIP NFE2L1 Primer2-R	CTAGGGCAAAACACAGAGGC
RIP NFE2L1 Primer3-F	TTTCCAGCAGACATTTCCAG
RIP NFE2L1 Primer3-R	CTGCATTTCCATGATGGACAT

F = Forward primer, R = Reverse primer.

Table S6. Primer sequences used for ChIP-qPCR.

Gene Name	Primer sequences 5'-3'
GPX4-F	GTCCCAGCTACTCGGGAAG
GPX4-R	GCAGAAAAGTG TCCCCAAC
GCLC-F	GTGGGGTGGGGTTGAAGATA
GCLC-R	AGCCTACCGTGGGGTGGGGT
G6PD-F	CTTTGGGGGAGTGCCAACAT
G6PD-R	ATCACAAGGGCCATGGGCTT
SRSF6-F	AGTTCTGCGGCTGGATTAGA
SRSF6-R	CTATATGGGCGGCCGGTG

F = Forward primer, R = Reverse primer.

Table S7. Oligonucleotide sequences used for dual-luciferase reporter assay.

Gene Name	Primer sequences 5'-3'
6×ARE-F	CTAGCCTGACTCAGCTATGACTCAGCTATGACTCAGCTATGACTCAGCTATGACTCAGCTATGACTCAGCCA
6×ARE-R	AGCTTGGCTGAGTCATAGCTGAGTCATAGCTGAGTCATAGCTGAGTCATAGCTGAGTCATAGCTGAGTCAGG

F = Forward primer, R = Reverse primer.

Table S8. Oligonucleotide sequences used for EMSA.

Gene Name	Primer sequences 5'-3'
ARE-F	ACTGAGGGTGACTCAGCAAAATC
ARE-R	TGACTCCCACTGAGTCGTTTTAG

F = Forward primer, R = Reverse primer.
